# Antibiotic Resistance, *spa* Typing and Clonal Analysis of Methicillin-Resistant *Staphylococcus aureus* (MRSA) Isolates from Blood of Patients Hospitalized in the Czech Republic

**DOI:** 10.3390/antibiotics10040395

**Published:** 2021-04-06

**Authors:** Katarina Pomorska, Vladislav Jakubu, Lucia Malisova, Marta Fridrichova, Martin Musilek, Helena Zemlickova

**Affiliations:** 1 Centre for Epidemiology and Microbiology, National Reference Laboratory for Antibiotics, National Institute of Public Health, 10000 Prague, Czech Republic; katarina.pomorska@szu.cz (K.P.); vladislav.jakubu@szu.cz (V.J.); lucia.malisova@szu.cz (L.M.); 2 Department of Clinical Microbiology, Faculty of Medicine and University Hospital, Charles University, 53002 Hradec Kralove, Czech Republic; 3 Department of Microbiology, 3rd Faculty of Medicine Charles University, University Hospital Kralovske Vinohrady, National Institute of Public Health, 10000 Prague, Czech Republic; marta.fridrichova@fnkv.cz; 4 Centre for Epidemiology and Microbiology, National Reference Laboratory for Meningococcal Infections, National Institute of Public Health, 10000 Prague, Czech Republic; martin.musilek@szu.cz

**Keywords:** *Staphylococcus aureus*, MRSA, *spa* typing, MLST, SCC*mec* typing, clonal analysis, epidemiology

## Abstract

*Staphylococcus aureus* is one of the major causes of bloodstream infections. The aim of our study was to characterize methicillin-resistant *Staphylococcus aureus* (MRSA) isolates from blood of patients hospitalized in the Czech Republic between 2016 and 2018. All MRSA strains were tested for antibiotic susceptibility, analyzed by *spa* typing and clustered using a Based Upon Repeat Pattern (BURP) algorithm. The representative isolates of the four most common *spa* types and representative isolates of all *spa* clonal complexes were further typed by multilocus sequence typing (MLST) and staphylococcal cassette chromosome *mec* (SCC*mec*) typing. The majority of MRSA strains were resistant to ciprofloxacin (94%), erythromycin (95.5%) and clindamycin (95.6%). Among the 618 strains analyzed, 52 different *spa* types were detected. BURP analysis divided them into six different clusters. The most common *spa* types were t003, t586, t014 and t002, all belonging to the CC5 (clonal complex). CC5 was the most abundant MLST CC of our study, comprising of 91.7% (n = 565) of *spa-*typeable isolates. Other CCs present in our study were CC398, CC22, CC8, CC45 and CC97. To our knowledge, this is the biggest nationwide study aimed at typing MRSA blood isolates from the Czech Republic.

## 1. Introduction

*Staphylococcus aureus* is an important opportunistic pathogen both in communities and in hospitals. It can cause broad spectrum of diseases, e.g., skin, soft tissue infections, heart, pleuropulmonary and osteoarticular infections [1]. It is considered to be one of the major causes of bloodstream infections (BSI) in Europe [2]. It was reported that a patient with bacteremia caused by methicillin-resistant *Staphylococcus aureus* (MRSA) is at a higher risk of all-cause mortality than a patient infected by methicillin-susceptible *Staphylococcus aureus* (MSSA) [3]. According to the European Antimicrobial Resistance Surveillance Network (EARS-Net) data, the proportion of MRSA isolates from blood from 2005 (until 2018) in the Czech Republic was around 14% (in the preceding years it was lower, from 4.3% to 8.5%) [4]. It is important to type MRSA isolates to get an insight into epidemiology, limit its possible spread or imply the infection control measures. Studies conducted over time engaged with typing Czech MRSA strains using different molecular methods such as multilocus sequence typing (MLST), staphylococcal cassette chromosome *mec* (SCC*mec*) typing, pulsed-field gel electrophoresis (PFGE) and ribotyping revealed clonal replacements. In 1996–1997 the most common MRSA clone was Brazilian clone (ST239, SCC*mec*IIIA, PFGE type B, ribotype H1) and Iberian clone (ST247, SCC*mec*IA, PFGE type A, ribotype H2) [5]. Around the year 2000, Brazilian clone was replaced by a unique “Czech clone”. They differed only in PFGE type and ribotype (F and H6 for the Czech clone, respectively) [6]. After 2001, epidemic clone EMRSA-15 (ST22, SCC*mec*IV) was detected increasingly [7]. Another clonal replacement was detected using staphylococcal protein A typing method (*spa* typing). Grundmann et al. [8] in his multicentric European study showed that the majority of Czech MRSA blood isolates from 2006–2007 period were typed as t003 (t003/ST225/SCC*mec*II). The retrospective typing of staphylococcal protein A gene of Czech MRSA blood isolates revealed that the clonal replacement took place in 2004, when the most common *spa* type t030 isolates were replaced by t003 isolates [9]. It was accompanied by the shift of antibiotic susceptibility of rifampicin and gentamicin [9]. Type t003 was the second most common MRSA *spa* type from bloodstream infections in 2011 in Europe [3]. As the previous studies have shown, the dominance of MRSA clones undergoes dynamic changes. The aim of our study was to type Czech MRSA blood isolates phenotypically (antibiotic susceptibility), genotypically (*spa* typing, MLST, SCC*mec* typing) and to infer their clonal relatedness by clustering them using Based Upon Repeat Pattern (BURP) algorithm.

## 2. Results

### 2.1. Antimicrobial Susceptibility of MRSA Strains

**Figure 1:**
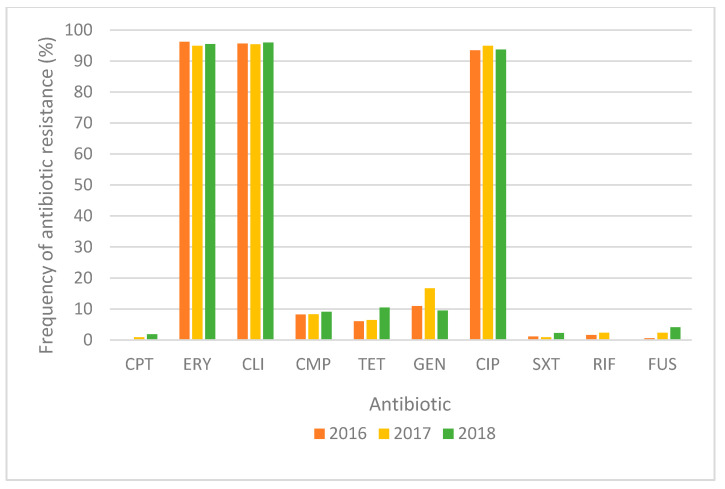
Frequency of antibiotic resistance (%) of the 618 single-patient MRSA blood isolates over the study period (2016–2018). CPT: ceftaroline, ERY: erythromycin, CLI: clindamycin, CMP: chloramphenicol, TET: tetracycline, GEN: gentamicin, CIP: ciprofloxacin, SXT: trimethoprim/sulfamethoxazole, RIF: rifampicin, FUS: fusidic acid. All the isolates were resistant to cefoxitine and susceptible to tigecycline, vancomycin and linezolid.

In total, in the 2016–2018 period, 618 single-patient MRSA blood isolates from the participating Czech EARS-Net laboratories were sent and analyzed in the National Reference Laboratory for Antibiotics (NRL for ATB), National Institute of Public Health (Prague, the Czech Republic). Resistance to methicillin (screened by cefoxitine disc [30 µg]) was confirmed by PCR for *mec* genes. All MRSA strains possessed an *mecA* gene (and were *mecC* negative). The majority of strains were resistant to erythromycin (n = 590; 95.5%), clindamycin (n = 591 (including 76 strains with inducible resistance); 95.6%), and ciprofloxacin (n = 581; 94.0%). Only 77 strains (12.5%) were resistant to gentamicin, 53 (8.6%) to chloramphenicol and 48 (7.8%) to tetracycline. The resistance to other antibiotics was rare: 15 strains (2.4%) were resistant to fusidic acid, 13 (2.1%) to rifampicin, 9 (1.5%) to trimethoprim/sulfamethoxazole and 6 (1.0%) to ceftaroline. All the confirmed MRSA isolates were susceptible to tigecycline, vancomycin and linezolid. The frequency of antibiotic resistance (%) over the study period is shown in Figure 1. The majority of strains (n = 600; 97.1%) were multidrug-resistant (MDR; i.e., non-susceptibility to at least one agent in three or more antimicrobial categories [10]). The most common MDR antibiotic resistance profile (resistance to cefoxitine, erythromycin, clindamycin and ciprofloxacin) was present in 429 (69.4%) strains. The more detailed characteristics of all the isolates from our study are shown in the Supplementary Table S1.

### 2.2. spa Typing, Cluster Analysis and Antibiotic Susceptibility within spa CCs

**Figure 2:**
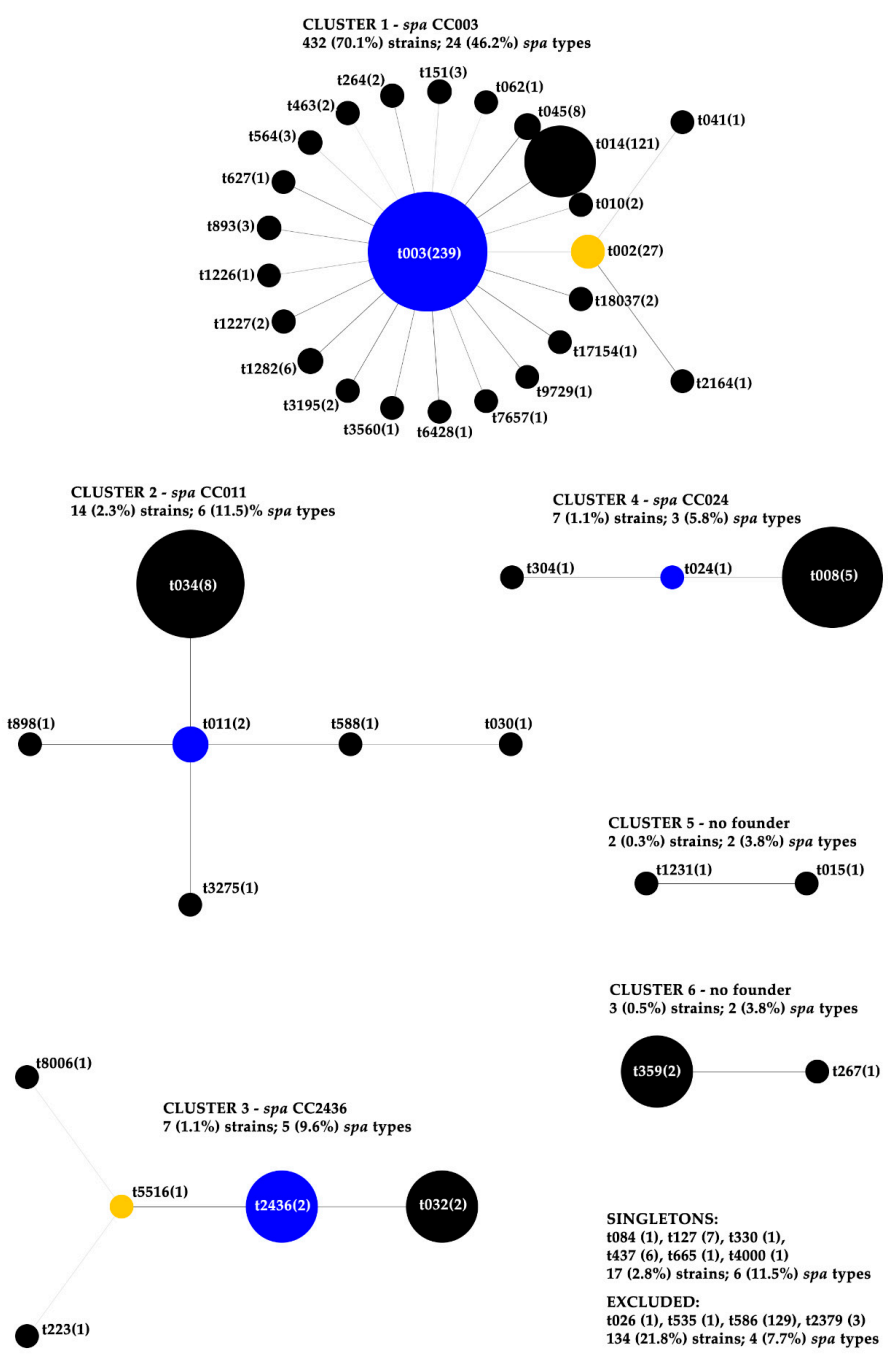
Based Upon Repeat Pattern (BURP) clustering of the *spa-*typed isolates. Isolates were clustered by Ridom Staph Type software using the following parameters: *spa* types were clustered if the cost was less or equal 6 and *spa* types that were shorter than 4 repeats were excluded from the analysis. The number in brackets represents number of isolates. The majority of the *spa* typeable isolates (75.5%) were grouped into 6 different clusters; 21.8% of the isolates were excluded from analysis and 2.8% of isolates were evaluated as singletons.

Altogether, 52 different *spa* types were detected in this study (Figure 2). Four *spa* types were dominant: t003 (n = 239; 38.8%), t586 (n = 129; 20.9%), t014 (n = 121; 19.6%) and t002 (n = 27; 4.4%). They were followed by t034 (n = 8; 1.3%), t045 (n = 8; 1.3%) and t127 (n = 7; 1.1%). Several *spa* types (n = 28; 53.8%) were detected only once. Two strains out of 618 (0.3%) were not typeable by *spa* typing.

The proportion of t003 and t002 did not differ much over the studied period (34.7–41.6% and 3.3–5.1%, respectively). The proportion of t586 isolates increased from 11.6% in 2016 to around 25% in the subsequent years. The proportion of t014 isolates decreased to 14.2% in 2018 (from more than 20% in the preceding years).

The majority of the *spa*-typeable isolates (n = 465; 75.5%) were grouped into 6 different clusters (4 *spa* CCs) (Figure 2). Cluster 5 and 6 did not have any founder and were not assigned into any *spa* CC (n = 5 strains; 0.8%). Several strains (n = 17; 2.8%) belonging to 6 different *spa* types were singletons. BURP algorithm excluded 134 (21.8%) strains from the analysis because of an inadequate number of repeats (Figure 2).

*spa* CC003 (cluster number 1) comprised of the greatest number of strains and the three most common *spa* types (t003, t002 and t014) (Figure 2). The majority of these strains were resistant to erythromycin, clindamycin and ciprofloxacin (Table 1). The second cluster, *spa* CC011, was the only cluster with a high proportion of tetracycline resistance (n = 13; 92.9%). The majority of isolates (>50%) within the two remaining *spa* CCs (2436 and 024) were resistant to erythromycin and ciprofloxacin. Five isolates (71.4%) from *spa* CC2436 were also clindamycin resistant; the resistance was inducible in 4 out of 5 aforementioned isolates. Gentamicin resistance (66.7%) was higher among isolates belonging to the cluster number 6 (no founder); however, this cluster did not consist of the representative number of isolates. The percentage of MDR strains and the number of resistances to different antibiotics for each *spa* CC is shown in Table 1.

### 2.3. spa Types and Resistance to Tetracycline

The high proportion of tetracycline resistance within *spa* CC011 prompted us to investigate its prevalence among the different *spa* types further. A high prevalence was detected in strains belonging to *spa* type t011 (n = 2; 100%), t034 (n = 7; 87.5%), t127 (n = 6; 85.7%) and t437 (n = 4; 66.7%). These *spa* types either belonged to *spa* CC011 (t011 and t034) or were classified as singletons (t127, t437). The four most common *spa* types, t586, t003, t014 and t002, had a low proportion of resistance to tetracycline: n = 13 (10.1%), n = 6 (2.5%), n = 3 (2.5%) and n = 0 (0%), respectively.

### 2.4. MLST, SCCmec Typing and MLST CCs in Relation to the Different spa CCs (BURP Clustering)

Representative isolates differing in antibiotic susceptibility profile of each *spa* CC (or cluster), together with singletons and strains excluded from BURP analysis, were further analyzed by MLST and SCC*mec* typing. Altogether, 40 isolates were typed: nine isolates belonging to *spa* cluster 1 (*spa* CC003), five isolates from cluster 2 (*spa* CC011), four isolates from cluster 3 (*spa* CC2436), three isolates from cluster 4 (*spa* CC024), two isolates belonging to cluster 5, three isolates from cluster 6, six isolates classified as singletons and eight strains excluded from BURP analysis because of inadequate number of repeats (Table 2).

In total, thirteen different MLST sequence types (STs) were typed among the 40 aforementioned isolates: ST1 (n = 1), ST5 (n = 2), ST8 (n = 3), ST22 (n = 4), ST45 (n = 4), ST59 (n = 1), ST72 (n = 1), ST97 (n = 3), ST225 (n = 13), ST398 (n = 5), ST1472 (n = 1), ST1535 (n = 1) and ST5688 (n = 1, new ST). They belonged to MLST CC1 (ST1), CC5 (ST225, ST5, ST5688), CC8 (ST8, ST72), CC15 (ST1535), CC22 (ST22), CC30 (ST1472), CC45 (ST45), CC59 (ST59), CC97 (ST97) and CC398 (ST398).

Strains were clustered in the same way either by BURP analysis or by Bionumerics software (MLST CC) with the exception of the two *spa* types. t4000 and t330 (classified as singletons by BURP analysis) belonged to MLST CC8 and CC45, respectively. For the more detailed relationship between different *spa* types and MLST CCs, see Table 2.

Four different SCC*mec* types were detected in our study. Isolates belonging to MLST CC5 were mainly typed as SCC*mec*II (15/16 isolates). Isolates belonging to other MLST CCs possessed either SCC*mec*IV, V or V_T_ type (Table 2).

### 2.5. Distribution of the Major spa Types and MLST CCs among the Czech Regions

**Figure 3:**
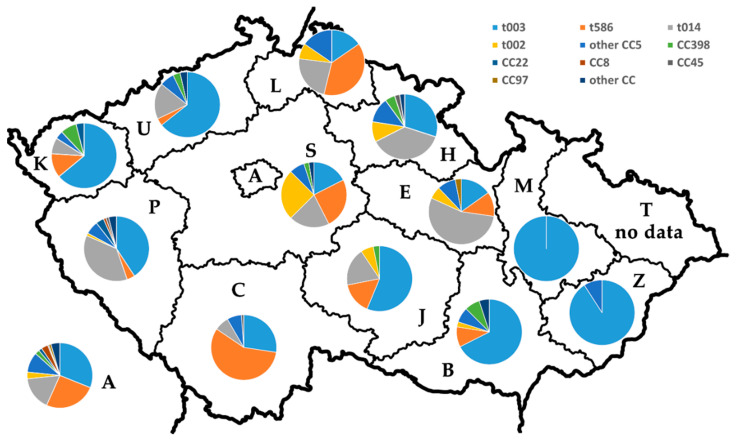
The distribution of the major *spa* types and MLST CCs among the Czech regions. A: Prague Region; S: Central Bohemian Region; C: South Bohemian Region; P: Pilsen Region; K: Karlovy Vary Region; U: Usti nad Labem Region; L: Liberec Region; H: Hradec Kralove Region; E: Pardubice Region; J: Vysocina Region; B: South Moravian Region; M: Olomouc Region; T: Moravian-Silesian Region; Z: Zlin Region.

Figure 3 and Table 3 show the distribution of the four major *spa* types and MLST CCs among the Czech regions. t003 strains were sent to NRL for ATB from all the Czech regions (n = 13; except the Moravian-Silesian, from which we did not obtain any MRSA strains). t003 was ubiquitous. It was dominant in the eastern part of the country: in the Olomouc region it was the only *spa* type detected and in Zlin region it represented more than 90% of the isolates. t586 strains (≥25% of the strains) were mainly isolated from the northwestern, southwestern and middle western part of the country. t014 strains were not detected in three regions (eastern/southeastern part of the country) and t002 strains in five regions.

## 3. Discussion

In our study we analyzed genotypically and phenotypically 618 MRSA strains isolated from blood of patients hospitalized in the Czech Republic in period 2016–2018. It represents 75% of the Czech MRSA isolates submitted to the EARS-Net.

We demonstrate that the majority of MRSA strains in our study are resistant to erythromycin, clindamycin and ciprofloxacin. A recent study from the Czech Republic also confirmed the high frequency of MRSA isolates resistant to antibiotics from the same classes (erythromycin, clindamycin and ofloxacin); however, only 4.5% of the studied MRSA isolates were derived from bloodstream infections (BSI) [11]. The earlier study concerning the antibiotic resistance of blood isolates from the Czech Republic (collection of MRSA blood isolates from 20 hospitals from the 2000–2002 period) reported resistance to more antibiotics (also gentamicin and rifampicin) [6]. This shift in a resistance phenotype is a result of clonal displacement (from ST239 to ST225 and ST5) [6,9].

We detected 52 different *spa* types among 616 *spa*-typeable MRSA strains. The most common *spa* types included t003, t586, t014 and t002, respectively. Type t003 is the most prevalent *spa* type from blood isolates in the Czech Republic from 2004 [9]. Our result concerning the high abundance of t003, t002 and t014 is in concordance with the multicentric European study that *spa*-typed MRSA blood isolates collected in 25 European countries, where t003 was reported to be the second most common, t002 the fourth and t014 the twentieth most common *spa* type [3]. High prevalence of t003, t586 and t014 was also detected in a recent Czech study investigating MRSA strains originating from various infections [11]. Neradova et al. detected a high proportion of t003, t002 and t014 from BSI from the Czech university hospital [12]. Types t003 and t014 were also detected to be the dominant *spa* types in a study investigating MRSA outbreak in the intensive care unit in the Czech tertiary care hospital [13]. Interesting is the high proportion of t586 (20.9% of the isolates) in our study. According to the Ridom Spa server database [14], this *spa* type has been reported in many European countries (e.g., Germany, Netherlands, France, Belgium, Croatia, Norway, Spain); however, to our knowledge, there has not been any other country detecting such a high proportion of it. MRSA strains typed as t586 were isolated from blood of patients hospitalized in the Czech Republic [11]; however, this is the first study detecting it from blood on a nationwide scale.

The *spa* type distribution varied between the Czech regions. t003 was widespread, it was the most frequently isolated from the eastern part of the country. t586 isolates were frequently isolated from northwestern, southwestern and middle western part of the country (Liberec, Prague, Central Bohemian and South Bohemian regions). Our results corroborate the observation of the recent study conducted by Tkadlec et al. [11] They typed MRSA isolated from various infection sites (or asymptomatic colonization) from 11 Czech hospitals. It is important to note that the dominance of just one *spa* type (or few) in some regions (our study) does not mean that the other *spa* types are not present in these parts of the country. The number of participating laboratories from different regions should be taken into consideration. Some laboratories participating in EARS-Net send only data, thus NRL for ATB does not obtain strains for further typing. From some regions we obtain strains from laboratories belonging to small healthcare facilities rather than laboratories from big hospitals. This might underrepresent prevalence of different *spa* types among the Czech regions in our study.

*spa*-typeable strains were divided into 6 clusters. Representative strains of each *spa* CC differing in antibiograms were typed by MLST and SCC*mec* typing. Our data analysis shows that the clustering results of BURP analysis and results from Bionumerics software (MLST CCs) are comparable. Isolates were clustered in the same way with either of the two aforementioned methods, with the exception of two *spa* types, which were evaluated as singletons by BURP analysis. Strommenger et al. [15] showed 96.8% concordance between the two methods (*spa* typing/BURP and MLST/eBURST).

Altogether, 565 (91.7%) MRSA strains from our study belonged to CC5. CC5 was reported to be the most abundant MLST CC of staphylococcal isolates from invasive infections in Europe, of which 80% were MRSA [16]. According to the results of more detailed genotyping of the representative strains, we can say that the most common genotype (clone) in our study was ST225/SCC*mec*II. This correlates with the results of whole-genome sequencing data of MRSA from invasive infections from Aanensen et al. [16] The geographic origin of this clone was Middle Europe, more exactly the Czech Republic and Germany. The European clade of ST225 is the descendant of the American clade. It diverged around 1995 and spread to several European countries (Germany, the Czech Republic, Switzerland and Denmark) [17]. Another clone present in our study, ST5/SCC*mec*II, was previously characterized as a USA100 clone (New York/Japan Clone) [18,19,20]. ST5 is an ancestor of ST225 with the variation in one MLST locus [17]. ST5-SCC*mec*II was reported also in other European countries, e.g., Hungary, Portugal or Austria [21,22,23]. We might hypothesize that the strain of genotype ST5688/SCC*mec*IV was derived from the Pediatric clone, which is characterized as ST5/SCC*mec*IV [24]. The difference between ST5 and ST5688 is only a single nucleotide in the internal fragment of the *pta* gene.

Our study demonstrates that livestock-associated MRSA (LA-MRSA) were isolated from the human bloodstream infections. The majority of the strains (92.9%) in the second cluster (*spa* CC011–MLST CC398) were resistant to tetracycline, which is a common marker of LA-MRSA [25]. All the t011 (*spa* CC 011) strains and the majority of the t034 (*spa* CC 011) and t127 strains (singletons) (87.5% and 85.7%, respectively) were resistant to the aforementioned antibiotic. The t011 and t034 isolates were typed as ST398 and possessed SCC*mec*IV or V element. The presence of the ST398 LA-MRSA strains and related *spa* types in the Czech Republic was confirmed by several studies. Tegegne et al. [26] reported a wide geographical spread of these strains (isolated from bulk tank milk of cows, sheep and goats) throughout the country. These *spa* types were detected in more than 90% of the *spa*-typed MRSA strains from the Czech livestock animals (pigs, cattle, goats and sheep) as well as from food of animal origin and the environment [27]. A recent study [28] investigated nasal MRSA carriage among veterinary professionals from the Czech Republic. The majority of isolates belonged to ST398 and were clustered into *spa* CC011. Tkadlec et al. [11] showed that 2.5% of the MRSA strains isolated from the various infections were of CC398. The results of our study show that LA-MRSA are also able to cause serious infections (bloodstream); however, the prevalence of these strains among MRSA isolated from blood remains low (2.3%). LA-MRSA ST398 could be the cause of a hospital outbreak, as reported by Wulf et al. [29].

EMRSA-15 (ST22/SCC*mec*IV) strains appeared in the early nineties and subsequently spread to the various hospitals in the United Kingdom and gradually disseminated to other European countries [30]. After 2001, this epidemic clone was detected in hospitals in the Czech Republic [7]. Our study demonstrates the presence of this clone among the isolates from BSI. Strains typed as ST22/SCC*mec*IV (*spa* types t032 and t2436) were grouped in the *spa* CC2436 (MLST CC22). Faria et al. [31] characterized MRSA isolated from BSI from Portugal, when the majority of them were typed as EMRSA-15. Another study [32] detected the presence of this clone in intensive care units from five different countries (years 2008–2011).

t008, t024, t304 and t4000 strains in our study (*spa* CC 024 and one singleton) belong to MLST CC8. The presence of t008 and t024 MRSA *spa* types in BSI in Europe is quite frequent: Grundman et al. [3] showed that t008 is the third most common and t024 is the 15th most common MRSA *spa* type isolated from blood of patients. Tkadlec et al. [11] detected CC8 strains (t008, t024, *etc.*) to be the second most frequently isolated MLST CC from MRSA infections of various origin from the 11 Czech hospitals. More than 64% of them were reported to be community associated (CA-MRSA).

Two isolates of t015 and t1231 (*spa* cluster 5), one t026 isolate (excluded from BURP analysis) and one t330 isolate (singleton) belong to MLST CC45. In 2006, *S. aureus* isolated from BSI typed as CC45 was one of the predominant clones circulating in Europe [16]. In our study we demonstrate the presence of the “Berlin IV” clone, which is characterized as ST45/SCC*mec*IV [33]. All the four aforementioned isolates were of the same genotype. This clone was also isolated from a nasal swab of the Czech MRSA carrier in 2008 [34]. This epidemic MRSA was initially isolated in Berlin hospitals in early nineties and subsequently disseminated to other areas of Germany [33,35]. A recent phylogenetic analysis proposes that acquisition of the SCC*mec*IV element occurred multiple times within the staphylococcal ST45 population [33].

The last *spa* cluster 6 in our study consisted of two t359 strains and one t267 strain. These isolates were typed as ST97 (MLST CC97). They possessed a SCC*mec*IV or SCC*mec*V element. CC97 MRSA strains were isolated from various infections (data from the Czech Republic, 0.9% of all the strains from the study) and their origin was CA-MRSA [11]. Studies have shown that ST97 MRSA are able to cause hospital outbreak [36,37]. 

Our study has typed a large collection of samples (75% of the Czech MRSA blood isolates submitted to EARS-Net in 2016–2018 period). Its limitation lies in a choice of the representative isolates belonging to the different *spa* CCs (for MLST analysis and SCC*mec* typing). Not all the *spa* types within some clonal complexes were further typed (for example, within the *spa* CC003 we further typed isolates belonging to the three most common *spa* types and no other rare *spa* types). This might underrepresent the presented diversity of genotypes.

Our study demonstrates that strains belonging to CC5 (ST225, ST5) are the most prevalent among MRSA isolated from the blood cultures from the Czech Republic. The majority of these strains confer a multidrug-resistant phenotype. Although other clones (e.g., EMRSA-15, Berlin IV) appear sporadically, CC5 clones remain the dominant MRSA bloodstream isolates from 2004.

## 4. Materials and Methods

### 4.1. Bacterial Strains

*S. aureus* strains isolated from blood of patients hospitalized in Czech hospitals in 2016–2018 were sent to the National Reference Laboratory for Antibiotics (NRL for ATB), National Institute of Public Health (Prague, the Czech Republic), by laboratories participating in EARS-Net, which is the largest publicly funded system for surveillance of antimicrobial resistance in Europe (https://www.ecdc.europa.eu/en/about-us/networks/disease-networks-and-laboratory-networks/ears-net-about, accessed on the 18 December 2020). At least two blood culture (BC) sets were taken and incubated in BC bottles in automatic systems for 5 days. Positive BC bottles were inoculated on blood agar plates and bacterial colonies were subsequently identified by Matrix—Assisted Laser Desorption Ionization—Time of Flight Mass Spectometry (MALDI-TOF) or other commonly used methods [38]. A total number of 1887 *S. aureus* isolates were reported to EARS-Net in 2016 (45 reporting laboratories), 1944 in 2017 (47 reporting laboratories) and 2244 in 2018 (48 reporting laboratories) (Table 4) [39]. Population sample representativeness, hospital sample representativeness and isolate representativeness was high during the studied period. Blood culture sets/1000 patient days was 18.0 in 2016 and 2017 and 17.0 in 2018 (Table 4) [39]. The staphylococcal isolates data are submitted into EARS-Net annually by the data manager on behalf of the participating laboratories, the majority of the isolates are regularly sent to NRL for ATB for confirmation and further typization. We obtained and analyzed 618 single-patient MRSA strains sent by 37 laboratories over the study period. This number represents 75% (n = 618/824) of all the MRSA strains submitted to the EARS-Net. In 2016 it was 69.2% (n = 182/263), 84% in 2017 (n = 216/257) and 72.4% in 2018 (n = 220/304) (Table 4) [39]. The estimated national population coverage included in the EARS-Net was 85% in 2016 and 2017. In 2018 it counted for 81% [39].

Isolates were inoculated on Nutrient Agar (OXOID, the Czech Republic) and cultivated overnight at 35 °C in aerobic atmosphere. Strain confirmation to the corresponding species was performed using MALDI-TOF (Microflex Bruker; Bremen, Germany) by flexControl software (Bruker Daltonics; Bremen, Germany).

### 4.2. Antibiotic Susceptibility Testing and MRSA Detection

Susceptibility to erythromycin, clindamycin, chloramphenicol, tigecycline, gentamicin, ciprofloxacin, trimethoprim/sulfamethoxazole, rifampicin, fusidic acid, vancomycin and linezolid was tested using broth microdilution method, while susceptibility to ceftaroline and tetracycline by disc diffusion method (according to the EUCAST methodology, breakpoints ver. 9.0—EUCAST 2019 [40]). Susceptibility to ceftaroline was tested using breakpoints for indications other than pneumonia (resistant <17mm, susceptible ≥20 mm). Inducible clindamycin resistance was tested by a broth microdilution method according to the CLSI methodology [41]. Methicillin resistance was screened using cefoxitine disc (30 µg). Strains with the zone diameter <22 mm were reported as MRSA.

### 4.3. Molecular Typing

#### 4.3.1. *mecA*/*mecC* Detection

The presence of genes encoding alternative penicillin-binding proteins (methicillin resistance) was confirmed by polymerase chain reaction (PCR) screening for *mecA*/*mecC* genes. *mecA* was detected using P4 (5′-TCC AGA TTA CAA CTT CAC CAG G-3′) and P7 (5′-CCA CTT CAT ATC TTG TAA CG-3′) primers [42]. PCR conditions were 4 min at 94 °C, followed by 30 cycles of 45 s at 94 °C, 45 s at 50 °C and 1 min at 72 °C. The final elongation was 2 min at 72 °C (Bio-Rad, DNA Engine Dyad^®^ Dual-Bay Thermal Cycler; Bio-Rad Laboratories, Hercules, California, USA). *mecC* gene was detected according to Stegger et al. [43]. All the primers used in our study are listed in the Supplementary Table S2.

#### 4.3.2. *spa* Typing and Based Upon Repeat Analysis (BURP)

In all MRSA isolates, a single locus of the repeat region X of the *S. aureus* protein A gene (*spa*) was sequenced. DNA amplification and DNA preparation for Sanger sequencing were performed according to the protocol from the official Ridom Spa Server website [44] using primers 1113f (5′-TAA AGA CGA TCC TTC GGT GAG C-3′) and 1514r (5′-CAG CAG TAG TGC CGT TTG CTT-3′). Sequences were evaluated and *spa* types determined using Ridom StaphType software.

To infer the clonal relatedness based on *spa* polymorphisms (*spa* CCs), MRSA strains were clustered by BURP analysis using Ridom StaphType software. Clustering parameters were chosen according to the RidomStaph Type user guide [45]: *spa* types were clustered if cost was less or equal 6 (value defining cluster dimension, the default value was used) and *spa* types that were shorter than 4 repeats were excluded (to include the highest number of *spa* types, the least possible value recommended was used). The strains with inadequate number of repeats were excluded from BURP analysis.

#### 4.3.3. Multilocus Sequence Typing (MLST)

MLST was performed as described by Enright et al. [46] The sequences of the seven approximately 450 bp long internal fragments of the housekeeping genes (carbamate kinase—*arcC*, shikimate dehydrogenase—*aroE*, glycerol kinase—*glpF*, guanylate kinase—*gmk*, phosphate acetyltransferase—*pta*, triosephosphate isomerase—*tpi* and acetyl coenzyme A acetyltransferase—*yqiL*) were amplified by PCR and sequenced by Sanger sequencing. The assignment of analyzed sequences and the determination of sequence types (STs) and clonal complexes (CCs) was done by Bionumerics software (ver. 7.6).

#### 4.3.4. SCC*mec* Typing

The assignment of SCC*mec* elements was performed using multiplex PCR according to the protocol employed by Milheiriço et al. [47] SCC*mec* types II and V (untypeable by the aforementioned method) were determined using an alternative set of primers (Supplementary Table S2) according to Zhang et al. [48] DNA sequence of SCC*mec* type V_T_ isolates was amplified using primers for SCC*mec* type V: Type V-F (5′-GAA CAT TGT TAC TTA AAT GAG CG-3′) and Type V-R (5′-TGA AAG TTG TAC CCT TGA CAC C-3′) [48]. PCR product of about 1600 bp was sequenced using Sanger sequencing. Sequence shared 100% identity with type V staphylococcal cassette chromosome of strain TSGH17 (AB512767.1) [49], which was reported as type V_T_ [50]. It was also identical with sequences of other V_T_ (VII) strains (AB478780.1, AB462393.1) [49,51].

## 5. Conclusions

To our knowledge, this study has typed (both phenotypically and genotypically) the largest collection of the Czech MRSA isolates from blood cultures so far. In general, MRSA strains were mainly resistant to erythromycin, clindamycin and ciprofloxacin. The majority of the isolates belonged to MLST CC5, with the most prevalent *spa* types t003, t586, t014 and t002. We have demonstrated that the dominant MRSA clone was ST225/SCC*mec*II. We confirmed the presence of LA-MRSA within strains grouped in the *spa* CC011 (CC398) as well as other MRSA clones.

## Figures and Tables

**Table 1:** Antibiotic resistance profiles of different *spa* clusters (clonal complexes) and multidrug-resistance within *spa* clusters.

Cluster	*spa* CC	No. (%) of Strains ^1^	No. (%) of Strains Resistant to Antibiotics ^2^										No. (%) of MDR Strains	No. of Resistant ATB (Mean Value) ^3^
			CPT	ERY	CLI	CMP	TET	GEN	CIP	SXT	RIF	FUS		
1	003	432 (70.1)	5 (1.2)	426 (98.6)	426 (98.6)	36 (8.3)	10 (2.3)	35 (8.1)	428 (99.1)	6 (1.4)	8 (1.9)	10 (2.3)	427 (98.8)	4.2
2	011	14 (2.3)	0	7 (50.0)	12 (85.7)	0	13 (92.9)	2 (14.3)	6 (42.9)	1 (7.1)	1 (7.1)	0	14 (100)	4.0
3	2436	7 (1.1)	0	5 (71.4)	5 (71.4)	0	0	0	6 (85.7)	0	0	0	5 (71.4)	3.3
4	024	7 (1.1)	0	5 (71.4)	2 (28.6)	0	0	2 (28.6)	4 (57.1)	0	0	1 (14.3)	4 (57.1)	3
5	no founder	2 (0.3)	0	0	0	0	0	0	0	0	0	0	0	1
6	no founder	3 (0.5)	0	0	0	0	0	2 (66.7)	0	0	0	1 (33.3)	1 (33.3)	2

CC: clonal complex, MDR: multidrug-resistance, ATB: antibiotics, CPT: ceftaroline, ERY: erythromycin, CLI: clindamycin, CMP: chloramphenicol, TET: tetracycline, GEN: gentamicin, CIP: ciprofloxacin, SXT: trimethoprim/sulfamethoxazole, RIF: rifampicin, FUS: fusidic acid; ^1^ (%) are calculated from the total number of *spa*-typeable strains; ^2^ all the isolates were resistant to cefoxitine and susceptible to tigecycline, vancomycin and linezolid; ^3^ number of resistances to the different antibiotics including cefoxitine.

**Table 2:** Results of the more detailed genotyping of the representative isolates differing in antibiotic susceptibility profiles belonging to the different *spa* clusters, singletons or strains excluded from BURP analysis.

Strain	*spa* Type	*spa* Cluster	*spa* CC	MLST ST	MLST CC	*SCCmec* Type	Resistance to Antibiotics ^1^
B0040949	t003	1	003	225	CC5	II	CIP, GEN, ERY, CLI
B0041781	t003	1	003	225	CC5	II	CMP, CIP, RIF, ERY, CLI
B0047063	t003	1	003	225	CC5	II	CIP, ERY, CLI
B0034821	t586	excluded	excluded	225	CC5	II	CMP, CIP, GEN, ERY, CLI,
B0038966	t586	excluded	excluded	225	CC5	II	CIP, ERY, CLI
B0043165	t586	excluded	excluded	225	CC5	II	CIP, GEN, ERY, CLI, TET
B0037533	t014	1	003	225	CC5	II	CIP, GEN
B0040744	t014	1	003	225	CC5	II	CIP, GEN, ERY, CLI
B0047366	t014	1	003	225	CC5	II	CIP, ERY, CLI
B0037993	t002	1	003	5	CC5	II	CMP, CIP, GEN, ERY, CLI
B0040837	t002	1	003	5688	CC5	IV	-
B0042384	t002	1	003	5	CC5	II	CIP, ERY, CLI
B0033841	t535	excluded	excluded	225	CC5	II	CIP, ERY, CLI
B0039941	t2379	excluded	excluded	225	CC5	II	CIP, ERY, CLI
B0040619	t2379	excluded	excluded	225	CC5	II	CIP, ERY, CLI
B0047250	t2379	excluded	excluded	225	CC5	II	CIP, ERY, CLI
B0038416	t011	2	011	398	CC398	V	CIP, TET
B0046007	t011	2	011	398	CC398	IV	CIP, GEN, SXT, TET
B0037087	t034	2	011	398	CC398	V	CLI, TET
B0043212	t034	2	011	398	CC398	V	CIP, CLI, TET
B0044843	t034	2	011	398	CC398	V	ERY, CLI, TET
B0034866	t032	3	2436	22	CC22	IV	CIP
B0039472	t032	3	2436	22	CC22	IV	CIP, ERY, CLI
B0036602	t2436	3	2436	22	CC22	IV	CIP, ERY, CLI
B0040230	t2436	3	2436	22	CC22	IV	CIP, ERY, CLI
B0034699	t008	4	024	8	CC8	nt	ERY
B0043674	t008	4	024	8	CC8	IV	CIP, ERY
B0043848	t008	4	024	8	CC8	IV	CIP, ERY, CLI
B0045550	t4000	singleton	singleton	72	CC8	nt	GEN, FUS
B0043746	t015	5	no founder	45	CC45	IV	-
B0044462	t1231	5	no founder	45	CC45	IV	-
B0032812	t026	excluded	excluded	45	CC45	IV	-
B0033429	t330	singleton	singleton	45	CC45	IV	ERY, CLI
B0040776	t267	6	no founder	97	CC97	V	GEN
B0037227	t359	6	no founder	97	CC97	V	GEN, FUS
B0048151	t359	6	no founder	97	CC97	IV	-
B0048051	t084	singleton	singleton	1535	CC15	V	GEN, FUS, TET
B0042118	t127	singleton	singleton	1	CC1	IV	ERY, CLI, TET
B0040853	t437	singleton	singleton	59	CC59	V_T_	CMP, ERY, CLI, TET
B0033532	t665	singleton	singleton	1472	CC30	IV	CIP, ERY, TET

CC: clonal complex, ST: sequence type, ERY: erythromycin, CLI: clindamycin, CMP: chloramphenicol, TET: tetracycline, GEN: gentamicin, CIP: ciprofloxacin, SXT: trimethoprim/sulfamethoxazole, RIF: rifampicin, FUS: fusidic acid; ^1^ cefoxitine is not listed in the antibiotic resistance profiles, since all the isolates were resistant.

**Table 3:** The total number of participating laboratories, isolates and distribution of the major *spa* types and strains belonging to the different MLST CCs among the Czech regions.

Region	No. of Particip. Laboratories	No. of Isolates ^1^	No. of *spa* Types	No. (%) of Isolates										
						CC5			CC398	CC22	CC8	CC45	CC97	Other CCs
				t003	t586	t014	t002	Other						
Prague	9	180	29	56 (31.1)	46 (25.6)	30 (16.7)	6 (3.3)	18 (10)	4 (2.2)	3 (1.7)	6 (3.3)	1 (0.6)	2 (1.1)	8 (4.4)
Central Bohemian	4	40	8	7 (17.5)	10 (25)	8 (20)	10 (25)	3 (7.5)	1 (2.5)	0	0	0	0	1 (2.5)
South Bohemian	4	84	9	23 (27.4)	48 (57.1)	6 (7.1)	0	6 (7.1)	0	0	0	1 (1.2)	0	0
Pilsen	2	76	15	31 (40.8)	3 (3.9)	28 (36.8)	1 (1.3)	5 (6.6)	0	3 (3.9)	1 (1.3)	1 (1.3)	0	3 (3.9)
Karlovy Vary	1	25	7	16 (64)	3 (12)	2 (8)	0	1 (4)	2 (8)	1 (4)	0	0	0	0
Usti nad Labem	2	28	7	18 (64.2)	1 (3.6)	5 (17.8)	0	2 (7.1)	1 (3.6)	0	0	0	0	1 (3.6)
Liberec	1	13	5	2 (15.4)	5 (38.5)	3 (23)	1 (7.7)	2 (15.4)	0	0	0	0	0	0
Hradec Kralove	3	40	9	12 (30)	0	15 (37.5)	4 (10)	5 (12.5)	2 (5)	0	0	1 (2.5)	0	1 (2.5)
Pardubice	3	33	8	5 (15.2)	4 (12.1)	18 (54.5)	2 (6.06)	3 (9.1)	0	0	0	0	1 (3.0)	0
Vysocina	4	32	5	18 (56.3)	5 (15.6)	6 (18.8)	2 (6.2)	0	1 (3.1)	0	0	0	0	0
South Moravian	2	40	10	27 (67.5)	4 (10.0)	0	1 (2.5)	3 (7.5)	3 (7.5)	0	0	0	0	2 (5.0)
Olomouc	1	14	1	14 (100.0)	0	0	0	0	0	0	0	0	0	0
Zlin	1	11	2	10 (90.9)	0	0	0	1 (9.1)	0	0	0	0	0	0

CC: clonal complex; ^1^ number of isolates comprises only of *spa-*typeable isolates.

**Table 4:** Number of reported isolates and data on the sample representativeness according to EARS-Net and number of MRSA strains sent to NRL for ATB from the participating laboratories in EARS-Net.

		Year of the Study			Reference
		2016	2017	2018	
No. of reported staphylococcal isolates	EARS-Net	1887	1944	2244	[39]
No. of MRSA (%)		263 (13.9)	257 (13.2)	304 (13.6)	
No. of single-patient MRSA strains sent to NRL for ATB from the laboratories participating in EARS-Net (% are calculated from the number of MRSA isolates reported in EARS-Net)	NRL for ATB	182 (69.2)	216 (84.0)	220 (72.4)	this study
Number of participating laboratories in our study		31	37	36	
Population sample representativeness	EARS-Net	high	high	high	[39]
Hospital sample representativeness		high	high	high	
Isolate sample representativeness		high	high	high	
Blood culture sets/1000 patient days		18.0	18.0	17.0	
Estimated national population coverage (%)		85	85	81	

## Data Availability

The data presented in this study are available on request from the corresponding author.

## Supplementary Materials

The following are available online at https://www.mdpi.com/article/10.3390/antibiotics10040395/s1, Table S1: The characteristics of the 618 MRSA isolates from blood of patients hospitalized in the Czech Republic (2016–2018), Table S2: Primers used in the study.
