# Supplementary material for: Antibiotic Resistance, spa Typing and Clonal Analysis of Methicillin-Resistant Staphylococcus aureus (MRSA) Isolates from Blood of Patients Hospitalized in the Czech Republic
Source: Antibiotics (Basel). 2021 Apr 6;10(4):395. doi: 10.3390/antibiotics10040395 (PMC8067498; doi:10.3390/antibiotics10040395)
Supplement: Supplementary file 1 [file antibiotics-10-00395-s001.zip › Supplementary table S1.pdf]

Table S1: The characteristics of the 618 MRSA isolates from blood of patients hospitalized in the Czech Republic (2016-2018).

Strain	Age	Sex	Year	Region	MIC [mg/l]											[mm]			*spa* type	*spa* cluster	*spa* CC	MLST ST	MLST CC	SCC*mec* type
					TGC	LNZ	CMP	CIP	GEN	SXT	RIF	FUS	ERY	CLI	VAN	FOX	TET	CPT						
B0032680	83	M	2016	Prague	0.06	2	8	>16	0.25	0.06	≤0.016	0.06	>8	**0.125**	1	6	27	17	t002	1	003	-	CC5	-
B0032732	61	M	2016	Prague	0.125	2	8	>16	0.25	0.125	≤0.016	0.125	>8	>4	1	6	25	19	t003	1	003	-	CC5	-
B0032812	59	F	2016	Prague	0.06	2	8	0.25	0.25	≤0.03	≤0.016	0.125	0.25	0.125	1	15	26	21	t026	E	E	45	CC45	IV
B0032906	73	F	2016	Pardubice	0.125	2	8	>16	0.5	0.06	≤0.016	0.125	>8	>4	1	6	31	20	t564	1	003	-	CC5	-
B0032911	84	F	2016	Hradec Kralove	0.125	2	8	>16	0.5	0.06	≤0.016	0.125	>8	>4	1	6	26	20	t003	1	003	-	CC5	-
B0032956	68	M	2016	South Moravian	0.125	2	8	>16	0.5	0.06	≤0.016	0.06	>8	>4	1	6	26	18	t003	1	003	-	CC5	-
B0032983	73	M	2016	Prague	0.06	2	8	0.5	0.5	0.06	≤0.016	0.06	>8	0.125	1	11	26	23	t008	4	024	-	CC8	-
B0032986	76	F	2016	Prague	0.125	2	8	>16	0.5	0.06	≤0.016	0.125	>8	>4	1	6	30	20	t586	E	E	-	CC5	-
B0033004	74	F	2016	Prague	0.125	2	8	>16	0.5	0.06	≤0.016	0.25	>8	>4	1	6	26	21	t003	1	003	-	CC5	-
B0033046	37	M	2016	Zlin	0.125	2	8	>16	0.5	0.06	≤0.016	0.125	>8	>4	1	6	26	21	t003	1	003	-	CC5	-
B0033069	70	M	2016	Usti nad Labem	0.06	1	4	>16	1	0.06	≤0.016	0.125	>8	>4	1	16	30	23	t014	1	003	-	CC5	-
B0033072	80	M	2016	Prague	0.125	2	>64	>16	0.5	0.06	≤0.016	0.125	>8	>4	1	6	30	18	t014	1	003	-	CC5	-
B0033106	73	M	2016	Prague	0.125	2	8	>16	0.5	0.06	≤0.016	0.125	>8	>4	1	6	26	17	t003	1	003	-	CC5	-
B0033181	90	F	2016	South Bohemian	0.125	2	8	>16	0.5	0.06	≤0.016	0.125	>8	>4	1	6	26	20	t003	1	003	-	CC5	-
B0033199	98	M	2016	Pilsen	0.125	2	16	>16	0.5	0.06	≤0.016	0.125	>8	>4	2	6	26	20	t003	1	003	-	CC5	-
B0033212	68	M	2016	Prague	0.125	2	8	>16	>16	0.06	≤0.016	0.125	>8	>4	1	6	30	21	t014	1	003	-	CC5	-
B0033289	77	F	2016	Pilsen	0.125	2	8	>16	0.25	≤0.03	≤0.016	0.06	>8	>4	1	6	28	22	t003	1	003	-	CC5	-
B0033326	51	M	2016	Prague	0.125	2	8	>16	0.5	0.125	≤0.016	0.06	>8	>4	1	6	28	19	t014	1	003	-	CC5	-
B0033338	67	F	2016	South Bohemian	0.06	1	8	>16	16	0.06	≤0.016	≤0.03	>8	>4	1	6	31	21	nt	-	-	-	-	-
B0033369	74	M	2016	Pilsen	0.06	2	8	>16	0.5	0.06	≤0.016	0.125	>8	>4	1	6	25	19	t014	1	003	-	CC5	-
B0033371	72	M	2016	Hradec Kralove	0.125	2	8	0.5	0.5	>4	≤0.016	0.06	0.25	0.125	1	6	26	20	t437	S	S	-	CC59	-
B0033382	79	M	2016	South Moravian	0.06	1	4	>16	0.5	0.06	≤0.016	0.06	>8	>4	1	6	27	19	t003	1	003	-	CC5	-
B0033389	84	M	2016	Prague	0.125	2	8	>16	0.5	≤0.03	≤0.016	0.06	>8	>4	1	6	26	19	t003	1	003	-	CC5	-
B0033428	78	M	2016	Pilsen	0.125	2	8	>16	0.5	0.06	≤0.016	0.06	>8	>4	1	6	26	20	t003	1	003	-	CC5	-
B0033429	14	F	2016	Pilsen	0.06	2	8	0.5	0.5	0.06	≤0.016	0.125	>8	**0.25**	1	14	25	23	t330	S	S	45	CC45	IV
B0033456	52	M	2016	Prague	0.125	2	8	>16	0.5	≤0.03	≤0.016	0.06	>8	>4	1	6	26	20	t003	1	003	-	CC5	-
B0033492	69	M	2016	Karlovy Vary	0.06	2	8	>16	0.5	0.125	≤0.016	0.125	>8	>4	1	6	28	23	t003	1	003	-	CC5	-
B0033532	0	M	2016	South Moravian	0.125	2	8	2	1	0.06	≤0.016	0.125	>8	0.25	2	11	14	22	t665	S	S	1472	CC30	IV
B0033533	65	M	2016	South Moravian	0.06	2	8	>16	0.5	≤0.03	≤0.016	0.06	>8	>4	1	6	27	17	t627	1	003	-	CC5	-
B0033535	64	M	2016	South Moravian	0.06	2	8	>16	0.25	0.06	≤0.016	0.06	>8	>4	1	6	24	18	t003	1	003	-	CC5	-
B0033574	79	M	2016	Prague	0.06	2	8	>16	0.5	0.06	≤0.016	0.125	>8	>4	1	6	27	18	t014	1	003	-	CC5	-
B0033576	64	F	2016	Prague	0.125	2	64	>16	0.25	0.06	≤0.016	0.125	>8	>4	1	6	27	19	t014	1	003	-	CC5	-
B0033620	53	M	2016	Central Bohemian	0.125	2	8	>16	0.25	0.06	≤0.016	0.125	>8	>4	1	8	26	20	t564	1	003	-	CC5	-
B0033674	54	F	2016	Prague	0.125	2	8	>16	0.25	≤0.03	≤0.016	0.125	>8	>4	2	6	30	20	t586	E	E	-	CC5	-
B0033722	74	M	2016	Pilsen	0.125	2	8	>16	0.5	≤0.03	≤0.016	0.06	>8	>4	1	6	27	19	t1226	1	003	-	CC5	-

B0033745	81	F	2016	Pardubice	0.06	2	8	>16	1	0.06	≤0.016	0.06	>8	**0.25**	2	6	28	19	t014	1	003	-	CC5	-
B0033746	71	M	2016	Pardubice	0.125	2	8	>16	0.5	0.06	0.125	0.06	>8	**0.25**	2	6	28	18	t2164	1	003	-	CC5	-
B0033841	88	M	2016	Pilsen	0.125	2	8	>16	0.25	0.06	≤0.016	0.125	>8	>4	1	6	25	19	t535	E	E	225	CC5	II
B0033885	66	M	2016	Zlin	0.125	2	8	>16	0.5	0.06	0.06	≤0.03	>8	>4	2	6	30	21	t1227	1	003	-	CC5	-
B0033911	85	F	2016	Hradec Kralove	0.125	2	8	>16	0.5	0.06	≤0.016	0.06	>8	**0.25**	1	6	27	20	t151	1	003	-	CC5	-
B0033914	93	F	2016	South Moravian	0.125	2	16	>16	0.5	0.06	≤0.016	0.06	>8	>4	2	6	27	20	t003	1	003	-	CC5	-
B0033934	62	M	2016	Prague	0.125	2	8	>16	0.5	0.06	≤0.016	0.125	>8	>4	2	6	29	19	t9729	1	003	-	CC5	-
B0034008	70	M	2016	Pilsen	0.25	2	8	>8	0.5	≤0.125	0.008	0.125	>8	>4	1	6	28	20	t014	1	003	-	CC5	-
B0034069	52	M	2016	Prague	0.25	2	8	>8	>8	0.25	>0.5	0.125	>8	>4	1	6	11	18	t030	2	011	-	CC398	-
B0034112	87	M	2016	Central Bohemian	0.25	2	8	>8	0.5	≤0.125	0.008	0.125	>8	>4	1	6	25	20	t003	1	003	-	CC5	-
B0034130	36	F	2016	South Moravian	0.25	2	8	0.5	0.5	1	≤0.004	0.125	0.5	>4	1	15	7	21	t034	2	011	-	CC398	-
B0034168	73	F	2016	Hradec Kralove	0.25	2	8	>8	0.5	≤0.125	0.008	0.125	>8	>4	1	6	28	19	t014	1	003	-	CC5	-
B0034260	81	F	2016	Prague	0.125	2	8	>8	>8	4	≤0.004	0.125	>8	>4	2	6	28	20	t014	1	003	-	CC5	-
B0034261	81	M	2016	Prague	0.125	2	32	>8	0.25	≤0.125	0.008	0.125	>8	>4	1	6	29	20	t014	1	003	-	CC5	-
B0034355	72	F	2016	Prague	0.25	2	8	0.5	0.5	≤0.125	0.008	0.06	>8	>4	1	6	10	21	t127	S	S	-	CC1	-
B0034404	59	M	2016	Hradec Kralove	0.125	2	8	>8	1	≤0.125	0.008	0.125	>8	**0.25**	1	6	26	17	t002	1	003	-	CC5	-
B0034411	66	M	2016	Prague	0.25	2	8	>8	1	≤0.125	0.008	0.125	>8	>4	2	6	27	22	t014	1	003	-	CC5	-
B0034413	82	M	2016	Central Bohemian	0.25	2	8	>8	0.5	≤0.125	0.008	0.25	>8	>4	1	6	26	19	t003	1	003	-	CC5	-
B0034431	54	F	2016	Hradec Kralove	0.25	2	8	>8	0.25	≤0.125	0.008	0.125	>8	**0.25**	1	6	25	20	t151	1	003	-	CC5	-
B0034439	72	M	2016	Hradec Kralove	0.25	2	8	>8	0.25	≤0.125	0.008	0.125	>8	>4	1	6	24	18	t014	1	003	-	CC5	-
B0034475	68	M	2016	Vysocina	0.125	2	8	>8	0.25	≤0.125	0.008	0.125	>8	**0.25**	1	6	25	19	t014	1	003	-	CC5	-
B0034480	85	F	2016	Pilsen	0.125	2	8	>8	0.5	≤0.125	0.008	0.125	>8	>4	1	9	25	20	t003	1	003	-	CC5	-
B0034509	76	M	2016	Prague	0.25	2	8	>8	1	≤0.125	0.008	0.125	>8	**0.25**	2	6	28	21	t002	1	003	-	CC5	-
B0034513	55	F	2016	Prague	0.25	2	8	>8	>8	0.5	0.008	0.125	>8	>4	1	6	26	19	t014	1	003	-	CC5	-
B0034562	66	M	2016	Prague	0.125	2	8	>8	>8	≤0.125	≤0.004	0.125	>8	**0.125**	1	16	26	21	t014	1	003	-	CC5	-
B0034615	75	M	2016	Zlin	0.125	2	8	>8	0.5	≤0.125	≤0.004	0.125	>8	>4	1	17	27	22	t003	1	003	-	CC5	-
B0034631	69	F	2016	South Moravian	0.25	2	8	>8	0.5	≤0.125	0.008	0.125	>8	>4	1	9	22	18	t003	1	003	-	CC5	-
B0034659	67	M	2016	South Bohemian	0.25	2	8	>8	0.5	≤0.125	0.008	0.125	>8	>4	1	6	26	21	t045	1	003	-	CC5	-
B0034699	60	F	2016	Pilsen	0.25	2	8	0.25	1	≤0.125	0.008	0.125	>8	0.25	1	16	27	21	t008	4	024	8	CC8	nt
B0034707	67	M	2016	South Bohemian	0.25	2	8	>8	0.25	≤0.125	0.008	0.125	>8	>4	1	6	26	19	t586	E	E	-	CC5	-
B0034713	37	M	2016	South Bohemian	0.25	2	16	>8	0.25	≤0.125	0.008	0.125	>8	>4	1	6	26	20	t586	E	E	-	CC5	-
B0034760	94	M	2016	Liberec	0.25	2	8	>8	0.5	≤0.125	0.008	0.06	>8	>4	1	9	30	19	t014	1	003	-	CC5	-
B0034821	71	M	2016	Prague	0.125	2	>64	>8	>8	0.25	≤0.004	0.125	>8	>4	1	6	32	19	t586	E	E	225	CC5	II
B0034866	81	M	2016	Prague	0.25	2	8	>8	0.5	≤0.125	0.008	0.125	0.5	0.25	1	6	30	18	t032	3	2436	22	CC22	IV
B0034896	58	F	2016	Pilsen	0.125	2	8	>8	0.5	≤0.125	0.008	0.25	>8	>4	1	6	31	20	t014	1	003	-	CC5	-
B0034897	64	F	2016	Pilsen	0.25	2	8	>8	1	≤0.125	0.008	0.125	>8	**0.25**	1	6	28	19	t5516	3	2436	-	CC22	-
B0034898	81	F	2016	Pilsen	0.25	0.5	8	>8	0.5	≤0.125	0.008	0.125	>8	>4	1	6	31	19	t014	1	003	-	CC5	-
B0034905	75	M	2016	Prague	0.25	2	8	>8	0.5	≤0.125	0.008	0.125	>8	>4	1	6	9	21	t003	1	003	-	CC5	-

B0034917	87	M	2016	Central Bohemian	0.125	2	16	>8	0.5	≤0.125	0.008	0.125	>8	>4	1	6	31	19	t564	1	003	-	CC5	-
B0034918	55	M	2016	Prague	0.25	2	8	>8	0.5	≤0.125	0.008	0.25	>8	>4	1	6	30	18	t1282	1	003	-	CC5	-
B0034947	78	M	2016	Hradec Kralove	0.25	2	8	>8	0.5	≤0.125	0.008	0.06	>8	>4	2	6	32	20	t003	1	003	-	CC5	-
B0034961	81	M	2016	Prague	0.125	2	8	>8	0.5	≤0.125	≤0.004	0.06	>8	>4	2	6	32	21	t014	1	003	-	CC5	-
B0034970	59	M	2016	Prague	0.25	2	8	>8	0.5	≤0.125	0.008	0.125	>8	>4	2	6	34	21	t586	E	E	-	CC5	-
B0035018	75	F	2016	Zlin	0.125	2	8	>8	0.5	≤0.125	0.008	0.125	>8	>4	1	6	24	21	t003	1	003	-	CC5	-
B0035026	66	M	2016	Liberec	0.25	2	16	>8	0.5	≤0.125	0.008	0.125	>8	>4	1	6	30	21	t014	1	003	-	CC5	-
B0035210	63	M	2016	South Bohemian	0.125	1	8	>8	0.25	≤0.125	0.016	0.125	>8	>4	1	9	26	21	t003	1	003	-	CC5	-
B0035212	65	M	2016	South Bohemian	0.125	2	8	>8	>8	0.25	≤0.004	0.125	>8	>4	1	6	28	18	t586	E	E	-	CC5	-
B0035213	93	F	2016	South Bohemian	0.125	2	8	>8	0.5	≤0.125	0.008	0.125	>8	>4	1	6	29	20	t003	1	003	-	CC5	-
B0035227	5	F	2016	Prague	0.25	2	8	>8	>8	≤0.125	≤0.004	0.06	>8	>4	1	6	30	19	t024	4	024	-	CC8	-
B0035230	74	M	2016	Prague	0.25	2	8	>8	0.5	≤0.125	0.008	0.125	>8	>4	2	6	29	19	t003	1	003	-	CC5	-
B0035236	65	M	2016	Prague	0.125	2	>64	>8	>8	0.25	≤0.004	0.125	>8	>4	1	6	28	19	t586	E	E	-	CC5	-
B0035242	82	M	2016	Vysocina	0.25	2	8	>8	0.5	≤0.125	≤0.004	0.125	>8	>4	1	6	27	21	t003	1	003	-	CC5	-
B0035269	13	M	2016	Hradec Kralove	0.25	2	8	>8	0.5	≤0.125	0.008	0.125	>8	>4	1	6	27	20	t003	1	003	-	CC5	-
B0035293	53	M	2016	Hradec Kralove	0.25	2	8	>8	0.5	≤0.125	0.008	0.125	>8	>4	1	6	28	20	t003	1	003	-	CC5	-
B0035306	69	F	2016	Prague	0.25	2	8	>8	0.5	≤0.125	≤0.004	0.25	>8	>4	1	6	27	20	t014	1	003	-	CC5	-
B0035392	60	F	2016	South Bohemian	0.25	2	8	>8	0.25	≤0.125	0.008	0.25	>8	>4	1	6	29	20	t014	1	003	-	CC5	-
B0035454	87	M	2016	Usti nad Labem	0.125	2	8	>8	0.5	≤0.125	0.008	0.125	>8	>4	1	6	28	20	t045	1	003	-	CC5	-
B0035487	62	M	2016	Hradec Kralove	0.25	2	8	>8	0.5	0.5	≤0.004	0.25	>8	>4	1	9	32	21	t014	1	003	-	CC5	-
B0035499	84	M	2016	Liberec	0.25	2	16	>8	0.5	≤0.125	0.03	0.125	>8	>4	1	6	32	18	t045	1	003	-	CC5	-
B0035511	63	M	2016	Pilsen	0.25	2	8	>8	0.5	2	0.008	0.25	>8	>4	1	6	29	21	t003	1	003	-	CC5	-
B0035519	51	M	2016	Vysocina	0.25	2	8	>8	0.5	≤0.125	0.008	0.25	>8	>4	1	6	27	19	t003	1	003	-	CC5	-
B0035633	20	M	2016	Central Bohemian	0.125	1	8	>8	>8	0.5	≤0.004	0.125	>8	>4	1	6	30	21	t003	1	003	-	CC5	-
B0035657	72	F	2016	Karlovy Vary	0.25	2	8	>8	0.5	≤0.125	0.008	0.125	>8	>4	1	6	30	20	t003	1	003	-	CC5	-
B0035661	87	F	2016	Karlovy Vary	0.25	2	8	>8	0.5	≤0.125	0.008	0.125	>8	>4	1	6	27	19	t003	1	003	-	CC5	-
B0035685	79	F	2016	Karlovy Vary	0.125	1	8	>8	>8	2	≤0.004	0.125	>8	>4	1	9	27	20	t003	1	003	-	CC5	-
B0035729	70	F	2016	Hradec Kralove	0.125	2	8	>8	0.5	0.25	≤0.004	0.125	>8	>4	1	6	28	19	t003	1	003	-	CC5	-
B0035738	82	F	2016	Usti nad Labem	0.25	2	8	>8	0.5	≤0.125	0.008	0.125	>8	>4	1	6	30	18	t17154	1	003	-	CC5	-
B0035820	82	F	2016	South Moravian	0.25	2	16	>8	0.5	≤0.125	0.008	0.25	>8	>4	1	6	29	19	t003	1	003	-	CC5	-
B0035860	65	F	2016	Prague	0.25	2	8	>8	0.25	≤0.125	>0.5	0.125	>8	>4	2	6	31	21	t014	1	003	-	CC5	-
B0035886	60	M	2016	Vysocina	0.25	2	8	>8	0.5	≤0.125	0.008	0.125	>8	>4	1	6	31	21	t003	1	003	-	CC5	-
B0035904	36	M	2016	Prague	0.125	2	8	>8	>8	1	≤0.004	0.125	>8	>4	1	6	34	19	t014	1	003	-	CC5	-
B0035972	71	F	2016	Vysocina	0.125	1	8	>8	1	≤0.125	0.008	0.125	>8	>4	1	6	30	19	t003	1	003	-	CC5	-
B0035974	80	F	2016	Prague	0.25	2	8	>8	1	≤0.125	0.008	0.125	>8	>4	1	6	32	20	t003	1	003	-	CC5	-
B0035986	91	F	2016	Prague	0.125	1	8	>8	1	≤0.125	0.008	0.125	>8	>4	1	6	29	19	t586	E	E	-	CC5	-
B0035996	73	F	2016	South Bohemian	0.125	1	8	>8	0.5	4	0.008	0.125	>8	>4	2	6	30	25	t463	1	003	-	CC5	-
B0036000	45	M	2016	South Bohemian	0.25	2	8	>8	0.5	4	≤0.004	0.125	>8	>4	1	14	27	25	t463	1	003	-	CC5	-

B0036009	70	M	2016	South Bohemian	0.25	2	8	>8	0.5	0.25	0.008	0.25	>8	>4	1	6	27	23	t003	1	003	-	CC5	-
B0036058	85	F	2016	South Moravian	0.25	2	16	>8	0.5	≤0.125	0.008	0.125	>8	>4	1	6	31	22	t003	1	003	-	CC5	-
B0036098	91	F	2016	Pilsen	0.25	2	8	>8	0.5	≤0.125	0.008	0.25	>8	>4	2	6	27	20	t003	1	003	-	CC5	-
B0036101	69	M	2016	Pilsen	0.25	2	8	>8	≤0.06	0.25	0.008	0.25	>8	>4	1	6	28	21	t014	1	003	-	CC5	-
B0036110	77	M	2016	Pardubice	0.125	2	8	>8	0.5	≤0.125	≤0.004	0.125	>8	**0.25**	1	6	29	19	t002	1	003	-	CC5	-
B0036123	61	M	2016	South Bohemian	0.125	2	8	>8	0.5	≤0.125	0.008	0.125	>8	>4	1	6	29	20	t003	1	003	-	CC5	-
B0036140	63	M	2016	South Moravian	0.125	1	8	>8	0.5	≤0.125	≤0.004	0.125	>8	>4	1	6	28	23	t003	1	003	-	CC5	-
B0036169	69	F	2016	Hradec Kralove	0.125	2	8	>8	0.5	≤0.125	0.008	0.125	>8	>4	1	6	32	21	t014	1	003	-	CC5	-
B0036259	58	M	2016	Prague	0.125	2	8	>8	0.5	≤0.125	0.008	0.25	>8	>4	1	6	27	20	t586	E	E	-	CC5	-
B0036296	75	M	2016	South Bohemian	0.125	2	8	>8	>8	≤0.125	≤0.004	0.125	>8	**0.125**	1	6	28	19	t014	1	003	-	CC5	-
B0036298	67	M	2016	South Bohemian	0.25	2	8	>8	0.5	≤0.125	0.008	0.125	>8	>4	1	6	29	19	t003	1	003	-	CC5	-
B0036317	48	M	2016	Pilsen	0.25	2	8	>8	0.5	≤0.125	≤0.004	0.25	>8	>4	1	6	11	23	t014	1	003	-	CC5	-
B0036371	89	M	2016	Olomouc	0.125	1	8	>8	0.5	≤0.125	≤0.004	0.125	>8	>4	1	6	28	21	t003	1	003	-	CC5	-
B0036381	68	M	2016	South Bohemian	0.125	2	8	>8	0.5	≤0.125	0.008	0.125	>8	>4	1	6	27	20	t003	1	003	-	CC5	-
B0036396	36	M	2016	Pilsen	0.125	2	8	>8	0.5	≤0.125	0.008	0.125	>8	>4	1	6	28	19	t014	1	003	-	CC5	-
B0036469	75	F	2016	Pilsen	0.25	1	8	>8	0.5	≤0.125	0.008	0.125	>8	>4	1	6	26	20	t014	1	003	-	CC5	-
B0036499	77	M	2016	Vysocina	0.125	1	8	>8	0.5	≤0.125	≤0.004	0.125	>8	>4	1	6	27	21	t003	1	003	-	CC5	-
B0036504	65	F	2016	Vysocina	0.125	2	8	>8	>8	≤0.125	≤0.004	0.125	>8	>4	1	6	30	19	t014	1	003	-	CC5	-
B0036519	72	M	2016	Usti nad Labem	0.25	2	8	>8	0.5	≤0.125	0.008	0.125	>8	>4	1	6	29	20	t014	1	003	-	CC5	-
B0036536	68	M	2016	Pardubice	0.125	1	8	>8	0.5	≤0.125	≤0.004	0.06	>8	>4	1	6	33	19	t003	1	003	-	CC5	-
B0036541	38	F	2016	Prague	0.125	2	8	>8	0.5	≤0.125	≤0.004	0.125	>8	>4	1	6	33	19	t586	E	E	-	CC5	-
B0036556	81	F	2016	Zlin	0.125	2	32	>8	0.5	≤0.125	0.008	0.125	>8	>4	1	6	27	19	t003	1	003	-	CC5	-
B0036588	92	M	2016	Prague	0.25	2	8	>8	0.5	≤0.125	0.008	0.125	>8	>4	1	6	31	18	t893	1	003	-	CC5	-
B0036600	53	M	2016	Olomouc	0.125	2	8	>8	1	0.25	0.008	0.25	>8	>4	1	6	29	18	t003	1	003	-	CC5	-
B0036602	59	M	2016	Pilsen	0.25	2	8	>8	1	≤0.125	0.008	0.25	>8	**0.125**	1	6	28	19	t2436	3	2436	22	CC22	IV
B0036614	76	M	2016	Central Bohemian	0.125	2	8	>8	>8	≤0.125	≤0.004	0.125	>8	>4	1	6	25	19	t014	1	003	-	CC5	-
B0036627	79	F	2016	Vysocina	0.25	2	8	>8	0.5	≤0.125	0.008	0.125	>8	**0.25**	1	6	28	19	t014	1	003	-	CC5	-
B0036640	60	M	2016	Vysocina	0.25	2	8	>8	0.5	≤0.125	≤0.004	0.125	>8	>4	1	6	30	18	t003	1	003	-	CC5	-
B0036718	55	M	2016	Central Bohemian	0.125	0.5	8	>8	0.5	≤0.125	0.008	0.125	>8	>4	1	6	31	21	t586	E	E	-	CC5	-
B0036765	33	M	2016	South Bohemian	0.125	2	8	>8	0.5	≤0.125	0.008	0.125	>8	>4	1	6	29	20	t586	E	E	-	CC5	-
B0036790	70	M	2016	Hradec Kralove	0.5	2	8	0.5	0.5	1	0.008	0.06	>8	>4	1	17	6	20	t034	2	011	-	CC398	-
B0036797	95	F	2016	South Moravian	0.125	1	8	>8	0.5	≤0.125	≤0.004	0.125	>8	>4	1	6	30	20	t003	1	003	-	CC5	-
B0036842	53	M	2016	Pilsen	0.25	2	8	0.5	0.5	≤0.125	≤0.004	0.125	>8	**0.125**	1	14	11	25	t127	S	S	-	CC1	-
B0036853	66	M	2016	Prague	0.125	1	8	>8	1	≤0.125	≤0.004	0.25	>8	>4	1	6	29	20	t586	E	E	-	CC5	-
B0036855	80	F	2016	Prague	0.125	1	8	>8	1	≤0.125	≤0.004	0.125	>8	>4	1	6	32	20	t003	1	003	-	CC5	-
B0036856	58	M	2016	Prague	0.125	1	8	>8	0.5	≤0.125	0.008	0.25	>8	>4	1	6	28	18	t586	E	E	-	CC5	-
B0036860	81	F	2016	Central Bohemian	0.125	2	16	>8	>8	≤0.125	0.008	0.125	>8	>4	1	6	30	20	t014	1	003	-	CC5	-
B0036907	63	M	2016	Pilsen	0.25	2	8	>8	0.5	≤0.125	0.008	0.125	>8	>4	1	6	11	21	t586	E	E	-	CC5	-

B0036948	80	M	2016	Karlovy Vary	0.125	1	8	>8	0.5	≤0.125	0.008	0.06	>8	>4	1	15	26	25	t034	2	011	-	CC398	-
B0036952	77	M	2016	Karlovy Vary	0.06	1	4	>8	0.5	≤0.125	≤0.004	0.06	>8	>4	1	15	29	23	t003	1	003	-	CC5	-
B0036958	78	F	2016	Karlovy Vary	0.5	2	8	>8	≤0.06	≤0.125	≤0.004	0.125	>8	>4	1	6	29	22	t003	1	003	-	CC5	-
B0036976	53	F	2016	Olomouc	0.25	2	8	>8	0.5	≤0.125	≤0.004	0.125	>8	>4	1	6	30	22	t003	1	003	-	CC5	-
B0037034	82	M	2016	Pilsen	0.125	2	8	>8	0.5	≤0.125	0.008	0.25	>8	>4	1	6	26	21	t586	E	E	-	CC5	-
B0037052	88	M	2016	South Bohemian	0.125	2	8	>8	0.5	≤0.125	0.008	0.125	>8	>4	1	6	32	22	t003	1	003	-	CC5	-
B0037053	75	M	2016	South Bohemian	0.25	2	8	>8	0.25	≤0.125	≤0.004	0.06	>8	>4	1	6	28	18	t003	1	003	-	CC5	-
B0037072	72	M	2016	Prague	0.125	1	8	>8	0.5	≤0.125	≤0.004	0.06	>8	>4	1	6	26	20	t586	E	E	-	CC5	-
B0037087	69	F	2016	Prague	0.25	2	8	0.5	1	0.5	≤0.004	0.125	0.5	>4	1	17	6	22	t034	2	011	398	CC398	V
B0037156	71	M	2016	Pilsen	0.125	2	8	>8	0.5	≤0.125	0.008	0.25	>8	>4	2	6	31	21	t003	1	003	-	CC5	-
B0037165	89	F	2016	South Bohemian	0.25	2	8	>8	0.25	≤0.125	≤0.004	0.125	>8	>4	1	6	30	20	t003	1	003	-	CC5	-
B0037186	72	M	2016	Prague	0.125	2	4	>8	0.5	0.25	0.008	0.125	>8	**0.125**	1	6	29	20	t003	1	003	-	CC5	-
B0037223	79	F	2016	Hradec Kralove	0.125	1	8	>8	0.5	≤0.125	0.008	0.125	>8	>4	1	6	28	20	t003	1	003	-	CC5	-
B0037224	73	F	2016	Hradec Kralove	0.125	2	8	>8	0.5	≤0.125	0.008	0.125	>8	**0.25**	1	6	28	20	t014	1	003	-	CC5	-
B0037227	64	M	2016	Prague	0.125	1	4	0.5	>8	≤0.125	≤0.004	4	0.5	0.125	1	16	27	24	t359	6	NF	97	CC97	V
B0037257	66	M	2016	Prague	0.125	2	8	>8	0.5	≤0.125	0.008	0.125	>8	**0.25**	1	6	25	19	t002	1	003	-	CC5	-
B0037293	50	M	2016	Prague	0.125	2	8	>8	0.5	0.25	0.008	0.125	>8	>4	1	6	28	22	t003	1	003	-	CC5	-
B0037323	79	M	2016	Prague	0.125	2	8	>8	0.5	0.25	≤0.004	0.5	>8	>4	1	15	26	23	t003	1	003	-	CC5	-
B0037343	80	M	2016	Prague	0.125	1	8	>8	0.5	≤0.125	≤0.004	0.125	>8	>4	1	6	27	20	t586	E	E	-	CC5	-
B0037402	74	M	2016	Olomouc	0.125	2	8	>8	0.5	≤0.125	0.008	0.125	>8	>4	1	6	27	20	t003	1	003	-	CC5	-
B0037413	78	F	2017	Pilsen	0.125	2	8	>8	≤0.06	≤0.125	≤0.004	0.25	>8	>4	1	6	30	20	t014	1	003	-	CC5	-
B0037414	51	M	2016	South Moravian	0.125	1	4	>8	0.125	≤0.125	>0.5	0.06	>8	>4	2	6	30	23	t003	1	003	-	CC5	-
B0037428	81	M	2016	Usti nad Labem	0.125	1	8	>8	0.5	≤0.125	≤0.004	0.125	>8	>4	1	6	27	20	t014	1	003	-	CC5	-
B0037438	65	M	2016	Pilsen	0.125	1	4	>8	0.5	≤0.125	≤0.004	0.125	>8	**0.125**	1	6	27	20	t014	1	003	-	CC5	-
B0037446	87	F	2016	Pilsen	0.125	2	8	>8	0.5	≤0.125	0.008	0.125	>8	>4	1	6	25	22	t014	1	003	-	CC5	-
B0037456	83	M	2016	Pilsen	0.125	1	8	>8	0.5	≤0.125	0.008	0.125	>8	>4	1	6	28	19	t014	1	003	-	CC5	-
B0037458	94	F	2016	Pilsen	0.125	1	4	0.5	0.5	≤0.125	0.008	0.125	0.5	0.125	1	6	12	20	t437	S	S	-	CC59	-
B0037459	38	F	2016	Pilsen	0.125	1	8	>8	0.5	≤0.125	≤0.004	0.06	>8	>4	1	6	30	24	t003	1	003	-	CC5	-
B0037504	84	F	2016	Vysocina	0.125	1	4	>8	>8	8	≤0.004	0.06	>8	**0.125**	1	6	30	20	t002	1	003	-	CC5	-
B0037529	69	M	2017	Vysocina	0.125	1	8	>8	0.5	≤0.125	≤0.004	0.125	>8	>4	1	6	27	21	t003	1	003	-	CC5	-
B0037532	61	F	2017	Prague	0.125	1	8	>8	0.25	≤0.125	0.008	0.125	>8	>4	1	6	29	21	t003	1	003	-	CC5	-
B0037533	85	M	2017	Central Bohemian	0.125	2	8	>8	>8	≤0.125	0.008	0.125	1	0.125	1	6	25	20	t014	1	003	225	CC5	II
B0037561	86	F	2016	Pilsen	0.125	2	8	>8	1	≤0.125	0.008	0.125	>8	>4	1	6	27	21	t003	1	003	-	CC5	-
B0037579	67	M	2017	Prague	0.25	2	8	>8	0.5	0.5	0.008	0.125	>8	**0.25**	2	6	26	19	t062	1	003	-	CC5	-
B0037602	79	F	2017	South Bohemian	0.125	2	8	>8	0.5	≤0.125	0.008	0.125	>8	>4	1	6	28	21	t003	1	003	-	CC5	-
B0037623	62	M	2017	Prague	0.125	2	8	>8	0.5	≤0.125	≤0.004	0.125	1	0.125	1	6	26	23	t003	1	003	-	CC5	-
B0037625	64	F	2017	South Moravian	0.125	1	8	>8	0.5	≤0.125	≤0.004	0.125	>8	>4	1	6	27	23	t003	1	003	-	CC5	-
B0037681	74	F	2017	Prague	0.125	1	8	>8	0.5	≤0.125	≤0.004	0.125	>8	>4	1	6	28	20	t586	E	E	-	CC5	-

B0037687	87	F	2016	South Bohemian	0.125	1	8	>8	>8	2	≤0.004	0.125	>8	>4	1	6	28	21	t586	E	E	-	CC5	-
B0037689	83	M	2016	South Bohemian	0.25	1	8	>8	0.5	≤0.125	0.008	0.125	>8	>4	1	6	27	19	t586	E	E	-	CC5	-
B0037703	60	M	2017	Vysocina	0.125	1	8	>8	0.5	≤0.125	0.008	0.125	>8	>4	1	6	28	22	t003	1	003	-	CC5	-
B0037709	83	F	2017	Prague	0.125	1	4	>8	0.5	≤0.125	0.008	0.125	>8	>4	1	6	26	20	t014	1	003	-	CC5	-
B0037711	58	F	2017	Liberec	0.125	1	8	>8	0.5	≤0.125	0.008	0.125	>8	>4	1	6	28	20	t586	E	E	-	CC5	-
B0037714	78	F	2017	Pardubice	0.125	2	8	>8	0.5	≤0.125	0.008	0.125	>8	>4	1	6	28	20	t014	1	003	-	CC5	-
B0037746	79	F	2016	South Moravian	0.25	2	8	>8	0.5	≤0.125	≤0.004	0.125	>8	>4	1	6	26	24	t003	1	003	-	CC5	-
B0037761	69	M	2017	Prague	0.125	2	8	>8	1	≤0.125	≤0.004	0.125	>8	>4	1	6	29	21	t586	E	E	-	CC5	-
B0037765	75	M	2017	Prague	0.125	2	8	>8	>8	≤0.125	≤0.004	0.125	>8	>4	1	6	29	21	t014	1	003	-	CC5	-
B0037793	69	M	2017	Prague	0.125	2	8	>8	0.5	≤0.125	≤0.004	0.125	>8	>4	1	6	32	18	t586	E	E	-	CC5	-
B0037804	63	F	2017	Prague	0.25	2	8	>8	0.5	≤0.125	0.25	0.25	>8	>4	2	6	32	22	t014	1	003	-	CC5	-
B0037807	53	M	2017	South Bohemian	0.25	2	8	>8	0.5	≤0.125	≤0.004	0.125	>8	>4	1	6	26	19	t003	1	003	-	CC5	-
B0037889	64	M	2017	Prague	0.25	2	8	>8	0.5	≤0.125	≤0.004	0.125	>8	>4	1	6	29	19	t003	1	003	-	CC5	-
B0037950	61	M	2017	Pardubice	0.125	2	8	>8	0.5	≤0.125	≤0.004	0.125	>8	>4	1	6	29	20	t586	E	E	-	CC5	-
B0037993	79	M	2017	Hradec Kralove	0.125	1	>64	>8	>8	≤0.125	≤0.004	0.125	>8	**0.25**	2	6	31	19	t002	1	003	5	CC5	II
B0038013	68	M	2017	Zlin	0.125	1	>64	>8	0.5	≤0.125	≤0.004	0.125	>8	>4	1	6	30	21	t003	1	003	-	CC5	-
B0038056	71	F	2017	Pilsen	0.125	1	8	>8	1	≤0.125	≤0.004	0.06	>8	>4	1	6	32	20	t014	1	003	-	CC5	-
B0038062	87	F	2017	Prague	0.125	1	4	0.5	0.25	≤0.125	≤0.004	0.06	>8	**0.125**	1	6	10	23	t127	S	S	-	CC1	-
B0038095	71	F	2017	Prague	0.125	2	8	>8	0.5	≤0.125	≤0.004	0.125	>8	>4	1	6	29	20	t586	E	E	-	CC5	-
B0038106	75	F	2017	Pardubice	0.125	2	8	>8	1	≤0.125	≤0.004	0.125	>8	>4	1	6	31	20	t014	1	003	-	CC5	-
B0038133	47	M	2017	Pilsen	0.25	2	16	>8	0.5	0.25	0.008	0.25	>8	>4	1	6	29	24	t014	1	003	-	CC5	-
B0038173	61	M	2017	Prague	0.125	2	8	>8	0.5	0.25	≤0.004	0.125	>8	>4	1	6	28	21	t003	1	003	-	CC5	-
B0038204	50	M	2017	Prague	0.125	1	8	>8	>8	≤0.125	≤0.004	0.125	>8	>4	1	6	34	21	t014	1	003	-	CC5	-
B0038207	66	F	2017	Prague	0.125	2	8	>8	0.5	≤0.125	≤0.004	0.125	>8	>4	1	6	30	21	t003	1	003	-	CC5	-
B0038212	52	M	2017	Pilsen	0.125	2	8	>8	1	≤0.125	≤0.004	0.125	>8	>4	1	6	31	21	t014	1	003	-	CC5	-
B0038218	66	M	2017	South Bohemian	0.125	2	8	>8	>8	0.25	≤0.004	0.125	>8	>4	1	6	30	19	t586	E	E	-	CC5	-
B0038219	77	F	2017	South Bohemian	0.125	1	8	>8	>8	0.25	≤0.004	0.125	>8	>4	1	6	11	19	t586	E	E	-	CC5	-
B0038224	75	M	2017	South Bohemian	0.125	1	8	>8	>8	≤0.125	≤0.004	0.125	>8	>4	2	6	29	19	t586	E	E	-	CC5	-
B0038225	69	M	2017	South Bohemian	0.125	1	8	>8	>8	0.25	≤0.004	0.125	>8	>4	1	6	30	19	t586	E	E	-	CC5	-
B0038236	79	M	2017	Prague	0.125	1	>64	>8	>8	0.25	≤0.004	0.125	>8	>4	1	6	31	20	t586	E	E	-	CC5	-
B0038254	81	F	2017	Prague	0.125	2	16	>8	1	≤0.125	≤0.004	0.125	>8	>4	1	6	33	21	t003	1	003	-	CC5	-
B0038324	72	F	2017	Pilsen	0.25	2	8	>8	1	≤0.125	0.008	0.125	>8	**0.25**	1	6	30	20	t014	1	003	-	CC5	-
B0038325	53	M	2017	Pilsen	0.125	1	4	>8	0.5	≤0.125	≤0.004	0.125	>8	**0.125**	1	6	39	20	t014	1	003	-	CC5	-
B0038358	78	M	2017	Hradec Kralove	0.125	1	8	>8	0.5	≤0.125	≤0.004	0.125	>8	>4	1	13	31	24	t003	1	003	-	CC5	-
B0038388	50	M	2016	Karlovy Vary	0.125	1	4	>8	0.5	≤0.125	≤0.004	0.06	>8	>4	1	6	34	22	t003	1	003	-	CC5	-
B0038407	77	M	2017	Karlovy Vary	0.125	1	8	>8	0.5	≤0.125	≤0.004	0.125	>8	>4	1	6	31	19	t586	E	E	-	CC5	-
B0038411	82	M	2017	Karlovy Vary	0.125	1	8	>8	0.25	≤0.125	≤0.004	0.125	>8	>4	1	6	33	19	t003	1	003	-	CC5	-
B0038416	63	M	2017	Karlovy Vary	0.25	1	4	2	0.5	≤0.125	≤0.004	0.06	0.5	0.125	1	15	6	24	t011	2	011	398	CC398	V

B0038426	89	M	2017	Pardubice	0.125	2	8	>8	0.5	≤0.125	≤0.004	0.125	>8	>4	1	6	28	22	t014	1	003	-	CC5	-
B0038503	82	F	2017	South Moravian	0.125	2	8	>8	1	≤0.125	0.008	0.06	>8	**0.125**	1	6	31	23	t002	1	003	-	CC5	-
B0038509	57	M	2017	Central Bohemian	0.125	1	4	0.25	0.5	2	≤0.004	0.06	0.5	>4	1	17	6	23	t898	2	011	-	CC398	-
B0038512	71	F	2017	Liberec	0.125	2	8	>8	0.5	≤0.125	0.008	2	>8	>4	1	6	31	21	t586	E	E	-	CC5	-
B0038541	54	M	2017	Prague	0.25	2	8	>8	1	≤0.125	≤0.004	0.125	>8	>4	2	6	30	21	t264	1	003	-	CC5	-
B0038546	85	F	2017	South Bohemian	0.125	2	8	>8	0.5	≤0.125	≤0.004	0.125	>8	**0.125**	1	6	33	19	t003	1	003	-	CC5	-
B0038549	82	M	2017	South Bohemian	0.125	1	8	>8	0.5	≤0.125	≤0.004	0.125	>8	>4	1	6	36	19	t586	E	E	-	CC5	-
B0038550	53	M	2017	South Bohemian	0.125	1	8	>8	0.25	≤0.125	0.008	0.125	>8	>4	1	6	34	21	t003	1	003	-	CC5	-
B0038582	57	F	2017	Prague	0.25	2	8	>8	0.5	≤0.125	0.008	0.25	>8	>4	1	6	28	21	t003	1	003	-	CC5	-
B0038591	85	M	2017	Prague	0.25	2	8	>8	0.5	0.5	≤0.004	0.125	>8	>4	1	6	31	22	t003	1	003	-	CC5	-
B0038691	63	M	2017	Hradec Kralove	0.25	2	8	>8	0.5	0.25	≤0.004	0.125	>8	>4	1	11	33	21	t014	1	003	-	CC5	-
B0038755	76	F	2017	Pilsen	0.25	2	8	>8	0.5	≤0.125	0.008	4	>8	>4	1	6	32	21	t003	1	003	-	CC5	-
B0038760	64	M	2017	Liberec	0.25	1	8	>8	0.5	0.25	≤0.004	0.125	>8	>4	1	9	29	22	t045	1	003	-	CC5	-
B0038815	91	F	2017	Pardubice	0.125	2	8	>8	0.5	≤0.125	≤0.004	0.25	>8	>4	1	6	29	19	t014	1	003	-	CC5	-
B0038821	73	M	2017	Prague	0.25	2	8	>8	1	≤0.125	≤0.004	0.25	>8	>4	1	6	33	24	t014	1	003	-	CC5	-
B0038842	73	F	2017	Usti nad Labem	0.25	2	8	>8	0.5	≤0.125	≤0.004	0.125	>8	>4	1	6	26	20	t014	1	003	-	CC5	-
B0038846	71	F	2017	Usti nad Labem	0.125	2	8	>8	0.5	≤0.125	≤0.004	0.125	>8	>4	1	6	28	21	t003	1	003	-	CC5	-
B0038849	71	F	2017	Usti nad Labem	0.125	2	8	>8	1	≤0.125	≤0.004	4	>8	>4	1	6	31	21	t003	1	003	-	CC5	-
B0038865	84	M	2017	Pilsen	0.125	2	>64	>8	>8	≤0.125	≤0.004	0.125	>8	**0.125**	1	6	29	20	t003	1	003	-	CC5	-
B0038958	64	M	2017	South Moravian	0.25	2	8	>8	0.5	≤0.125	≤0.004	0.125	>8	>4	1	6	29	22	t003	1	003	-	CC5	-
B0038965	60	F	2017	Prague	0.25	2	8	>8	0.5	≤0.125	0.008	0.25	>8	**0.125**	1	6	27	21	t003	1	003	-	CC5	-
B0038966	63	M	2017	Prague	0.25	2	8	>8	1	≤0.125	≤0.004	0.25	>8	>4	1	6	31	21	t586	E	E	225	CC5	II
B0038978	68	F	2017	Pilsen	0.25	2	8	>8	0.5	≤0.125	≤0.004	0.125	>8	>4	1	6	29	19	t003	1	003	-	CC5	-
B0039011	83	M	2017	Hradec Kralove	0.125	2	>64	>8	>8	≤0.125	≤0.004	0.125	>8	>4	1	6	32	20	t1282	1	003	-	CC5	-
B0039012	73	M	2017	Hradec Kralove	0.25	2	8	>8	0.5	≤0.125	≤0.004	0.125	>8	>4	1	6	31	20	t014	1	003	-	CC5	-
B0039026	63	M	2017	Prague	0.25	2	8	>8	1	0.25	0.008	0.25	>8	>4	1	6	28	21	t014	1	003	-	CC5	-
B0039041	66	F	2017	Prague	0.25	2	8	>8	0.5	≤0.125	0.008	0.125	>8	>4	1	8	26	19	t003	1	003	-	CC5	-
B0039059	56	M	2017	South Bohemian	0.25	2	8	>8	>8	16	≤0.004	0.125	>8	>4	1	6	30	18	t586	E	E	-	CC5	-
B0039060	63	M	2017	South Bohemian	0.25	1	8	>8	0.5	≤0.125	≤0.004	0.125	>8	>4	1	6	29	19	t586	E	E	-	CC5	-
B0039061	55	F	2017	South Bohemian	0.125	2	8	>8	>8	0.25	0.008	0.125	>8	>4	1	6	27	19	t586	E	E	-	CC5	-
B0039081	70	M	2017	Prague	0.25	2	8	>8	0.5	0.5	0.008	0.125	>8	>4	1	6	28	21	t003	1	003	-	CC5	-
B0039089	81	M	2017	Pilsen	0.25	2	8	>8	0.5	0.25	0.008	0.125	>8	>4	1	6	28	20	t003	1	003	-	CC5	-
B0039153	71	F	2017	Prague	0.125	2	8	>8	0.5	0.5	≤0.004	0.125	>8	>4	1	6	27	18	t003	1	003	-	CC5	-
B0039159	79	F	2017	Usti nad Labem	0.25	2	8	>8	0.5	≤0.125	≤0.004	0.125	>8	>4	1	21	28	22	t003	1	003	-	CC5	-
B0039205	44	M	2017	South Bohemian	0.5	2	8	>8	0.5	0.25	0.008	0.125	>8	>4	1	6	24	19	t586	E	E	-	CC5	-
B0039285	67	M	2017	Prague	0.125	2	8	>8	0.5	≤0.125	≤0.004	0.125	>8	**0.25**	1	6	27	21	t002	1	003	-	CC5	-
B0039287	91	F	2017	Prague	0.25	2	8	>8	0.5	≤0.125	0.008	0.25	>8	>4	1	6	27	20	t003	1	003	-	CC5	-
B0039300	87	F	2017	Pardubice	0.25	2	8	>8	1	≤0.125	≤0.004	0.125	>8	>4	1	6	27	19	t014	1	003	-	CC5	-

B0039326	83	F	2017	Central Bohemian	0.125	2	8	>8	0.5	≤0.125	0.008	0.125	>8	>4	1	6	27	19	t586	E	E	-	CC5	-
B0039411	76	M	2017	Prague	0.125	2	8	>8	0.5	≤0.125	0.008	0.125	>8	>4	1	8	27	24	t014	1	003	-	CC5	-
B0039440	70	M	2017	Prague	0.25	2	8	>8	0.25	≤0.125	≤0.004	0.125	>8	>4	1	11	29	20	t003	1	003	-	CC5	-
B0039441	78	M	2017	Prague	0.125	2	8	>8	>8	≤0.125	≤0.004	0.125	>8	>4	1	6	29	19	t586	E	E	-	CC5	-
B0039472	75	F	2017	Prague	0.25	2	8	>8	0.25	≤0.125	≤0.004	0.125	>8	>4	1	6	27	20	t032	3	2436	22	CC22	IV
B0039490	70	F	2017	Hradec Kralove	0.125	2	8	>8	0.5	0.25	≤0.004	0.25	>8	>4	1	9	25	22	t014	1	003	-	CC5	-
B0039530	87	M	2017	Prague	0.25	2	8	>8	0.5	≤0.125	0.008	0.125	>8	>4	1	6	25	19	t586	E	E	-	CC5	-
B0039553	79	M	2017	Pilsen	0.125	2	8	>8	0.5	≤0.125	≤0.004	0.125	>8	>4	1	6	24	19	t003	1	003	-	CC5	-
B0039600	46	F	2017	Central Bohemian	0.25	2	8	>8	>8	≤0.125	≤0.004	0.125	>8	>4	1	7	26	19	t014	1	003	-	CC5	-
B0039637	76	F	2017	Karlovy Vary	0.25	2	8	>8	0.5	≤0.125	≤0.004	0.125	>8	**0.25**	1	6	24	22	t8006	3	2436	-	CC22	-
B0039638	65	M	2017	Karlovy Vary	0.25	2	8	>8	0.5	≤0.125	0.008	0.125	>8	>4	1	6	28	24	t003	1	003	-	CC5	-
B0039645	43	F	2017	Prague	0.125	2	8	0.5	0.5	4	≤0.004	0.125	0.5	0.25	1	14	28	26	t223	3	2436	-	CC22	-
B0039707	67	F	2017	Prague	0.125	2	8	>8	0.25	0.25	≤0.004	0.06	>8	>4	1	19	31	26	t003	1	003	-	CC5	-
B0039769	82	M	2017	Usti nad Labem	0.125	1	8	>8	1	≤0.125	≤0.004	0.125	>8	>4	1	6	26	20	t014	1	003	-	CC5	-
B0039805	74	M	2017	Prague	0.125	1	8	>8	0.25	≤0.125	≤0.004	0.125	>8	>4	1	6	27	19	t586	E	E	-	CC5	-
B0039829	61	M	2017	Pilsen	0.125	2	8	>8	1	0.25	0.008	0.125	>8	**0.25**	1	6	25	21	t002	1	003	-	CC5	-
B0039856	94	M	2017	Olomouc	0.25	2	8	>8	0.5	≤0.125	0.008	0.125	>8	>4	1	6	25	20	t003	1	003	-	CC5	-
B0039869	80	F	2017	Pardubice	0.25	2	8	>8	0.5	≤0.125	0.008	0.125	>8	>4	1	7	26	23	t014	1	003	-	CC5	-
B0039941	73	F	2017	Prague	0.25	2	8	>8	0.5	≤0.125	≤0.004	0.125	>8	>4	1	6	26	20	t2379	E	E	225	CC5	II
B0040008	76	M	2017	Pilsen	0.125	1	8	>8	0.5	≤0.125	≤0.004	0.06	>8	>4	1	6	33	22	t003	1	003	-	CC5	-
B0040020	85	M	2017	South Moravian	0.125	1	8	>8	0.5	≤0.125	≤0.004	0.125	>8	>4	1	6	27	21	t003	1	003	-	CC5	-
B0040038	90	F	2017	South Bohemian	0.125	2	8	>8	0.25	≤0.125	≤0.004	0.06	>8	**0.125**	1	6	30	21	t014	1	003	-	CC5	-
B0040073	79	F	2017	Vysocina	0.25	2	8	>8	0.5	≤0.125	0.008	0.125	>8	>4	1	6	24	19	t586	E	E	-	CC5	-
B0040091	63	M	2017	Hradec Kralove	0.25	2	8	>8	1	0.25	0.008	0.125	>8	>4	1	6	27	20	t003	1	003	-	CC5	-
B0040100	74	M	2017	Prague	0.25	2	8	>8	0.5	0.25	0.008	0.125	>8	>4	1	12	23	24	t003	1	003	-	CC5	-
B0040164	62	M	2017	South Bohemian	0.125	2	8	>8	0.25	≤0.125	0.008	0.125	>8	**0.125**	1	6	32	21	t014	1	003	-	CC5	-
B0040177	76	F	2017	Hradec Kralove	0.125	2	8	>8	0.5	≤0.125	≤0.004	0.125	>8	>4	1	10	27	22	t014	1	003	-	CC5	-
B0040179	86	F	2017	Usti nad Labem	0.125	1	8	>8	0.5	≤0.125	≤0.004	0.125	>8	>4	1	6	31	22	t003	1	003	-	CC5	-
B0040189	68	M	2017	Zlin	0.125	2	8	>8	0.25	≤0.125	0.008	0.125	>8	>4	1	6	32	21	t003	1	003	-	CC5	-
B0040208	63	F	2017	Prague	0.125	2	8	>8	0.5	≤0.125	≤0.004	0.06	>8	>4	1	6	33	19	t014	1	003	-	CC5	-
B0040209	92	F	2017	Prague	0.125	2	8	>8	0.5	≤0.125	≤0.004	0.125	>8	**0.125**	1	6	30	20	t003	1	003	-	CC5	-
B0040210	77	F	2017	Prague	0.125	1	8	>8	>8	0.25	≤0.004	0.06	>8	>4	1	6	30	21	t003	1	003	-	CC5	-
B0040216	57	F	2017	South Bohemian	0.125	1	8	>8	0.25	≤0.125	0.008	0.125	>8	>4	1	6	30	20	t1282	1	003	-	CC5	-
B0040223	42	M	2017	Pardubice	0.125	2	8	>8	0.5	≤0.125	≤0.004	0.125	>8	>4	1	6	28	19	t586	E	E	-	CC5	-
B0040230	44	M	2017	Pilsen	0.06	2	8	>8	0.5	≤0.125	≤0.004	0.125	>8	**0.25**	1	13	27	27	t2436	3	2436	22	CC22	IV
B0040258	71	F	2017	South Bohemian	0.25	2	>64	>8	0.5	≤0.125	0.008	0.125	>8	>4	1	6	29	20	t586	E	E	-	CC5	-
B0040275	60	F	2017	Prague	0.125	2	8	1	0.5	≤0.125	≤0.004	0.06	0.5	0.125	1	15	30	22	t045	1	003	-	CC5	-
B0040317	65	M	2017	Pilsen	0.25	2	8	>8	0.5	≤0.125	≤0.004	0.125	>8	>4	1	6	32	21	t003	1	003	-	CC5	-

B0040333	53	M	2017	Prague	0.125	2	8	>8	0.5	≤0.125	≤0.004	0.125	>8	>4	1	6	29	23	t003	1	003	-	CC5	-
B0040334	61	F	2017	Hradec Kralove	0.125	1	64	>8	>8	≤0.125	≤0.004	0.06	>8	>4	1	6	29	20	t1282	1	003	-	CC5	-
B0040344	59	F	2017	Vysocina	0.125	1	8	>8	>8	0.5	≤0.004	0.06	>8	>4	2	6	30	18	t586	E	E	-	CC5	-
B0040376	76	M	2017	South Moravian	0.25	2	8	>8	0.25	≤0.125	≤0.004	0.125	>8	**0.125**	1	6	30	19	t586	E	E	-	CC5	-
B0040398	75	M	2017	Liberec	0.25	2	8	>8	1	0.25	0.008	0.125	>8	>4	1	6	30	20	t586	E	E	-	CC5	-
B0040444	68	F	2017	Pardubice	0.25	2	8	>8	0.25	≤0.125	≤0.004	0.125	>8	**0.125**	1	6	11	20	t586	E	E	-	CC5	-
B0040462	78	F	2017	Prague	0.25	2	16	>8	0.25	≤0.125	≤0.004	0.125	>8	>4	1	6	30	20	t014	1	003	-	CC5	-
B0040608	70	M	2017	Prague	0.125	2	8	>8	0.125	0.25	>0.5	0.125	>8	>4	1	6	28	24	t014	1	003	-	CC5	-
B0040619	50	M	2017	Pilsen	0.125	2	8	>8	0.25	≤0.125	0.008	0.125	>8	>4	1	6	29	22	t2379	E	E	225	CC5	II
B0040634	70	M	2017	Prague	0.25	2	8	>8	0.5	≤0.125	≤0.004	0.125	>8	>4	1	6	29	22	t003	1	003	-	CC5	-
B0040704	54	F	2017	Pilsen	0.5	2	16	>8	0.25	0.25	0.016	0.125	>8	>4	2	19	30	23	t3195	1	003	-	CC5	-
B0040711	80	M	2017	South Moravian	0.25	2	8	>8	0.5	≤0.125	0.008	0.125	>8	>4	1	6	31	19	t586	E	E	-	CC5	-
B0040720	77	M	2017	Hradec Kralove	0.125	2	8	>8	0.5	≤0.125	≤0.004	0.125	>8	>4	1	14	25	22	t014	1	003	-	CC5	-
B0040740	60	M	2017	Central Bohemian	0.25	2	8	>8	1	2	≤0.004	0.125	>8	>4	1	6	25	21	t003	1	003	-	CC5	-
B0040744	62	M	2017	Prague	0.125	1	4	>8	>8	0.25	≤0.004	0.125	>8	>4	1	17	24	23	t014	1	003	225	CC5	II
B0040768	87	F	2017	South Moravian	0.125	1	8	>8	0.25	≤0.125	≤0.004	0.125	>8	>4	1	6	29	20	t586	E	E	-	CC5	-
B0040772	75	M	2017	Hradec Kralove	0.25	2	8	>8	0.5	≤0.125	≤0.004	0.25	>8	>4	1	6	27	19	t003	1	003	-	CC5	-
B0040776	34	M	2017	Pardubice	0.25	2	8	1	>8	≤0.125	0.008	0.125	0.5	0.125	2	17	27	23	t267	6	NF	97	CC97	V
B0040788	57	M	2017	Prague	0.25	1	64	0.5	0.5	0.5	≤0.004	0.06	>8	>4	1	14	13	20	t437	S	S	-	CC59	-
B0040792	65	M	2017	South Bohemian	0.125	2	64	>8	>8	0.25	0.008	0.25	>8	>4	2	6	30	21	t586	E	E	-	CC5	-
B0040820	73	M	2017	Pilsen	0.25	2	8	>8	0.25	0.25	0.008	0.125	>8	>4	1	6	25	20	t003	1	003	-	CC5	-
B0040837	41	M	2017	Central Bohemian	0.25	2	8	0.5	0.5	≤0.125	0.008	0.125	0.5	0.125	1	10	26	21	t002	1	003	5688	CC5	IV
B0040853	64	F	2017	Prague	0.25	2	64	0.5	0.25	≤0.125	0.008	0.125	>8	>4	1	14	12	20	t437	S	S	59	CC59	V_T
B0040871	72	F	2017	Pardubice	0.25	2	8	>8	0.25	≤0.125	0.008	0.125	>8	**0.125**	1	6	25	17	t014	1	003	-	CC5	-
B0040931	86	M	2017	Karlovy Vary	0.25	2	8	>8	0.25	≤0.125	0.008	0.125	>8	>4	1	6	27	19	t003	1	003	-	CC5	-
B0040949	29	M	2017	Karlovy Vary	0.125	2	8	>8	>8	1	≤0.004	0.125	>8	>4	1	6	27	19	t003	1	003	225	CC5	II
B0041030	61	F	2017	Prague	0.25	2	8	>8	0.5	≤0.125	0.008	0.125	>8	**0.125**	1	6	28	19	t002	1	003	-	CC5	-
B0041037	87	M	2017	Hradec Kralove	0.25	2	8	>8	0.25	≤0.125	≤0.004	0.125	>8	**0.125**	1	14	27	21	t003	1	003	-	CC5	-
B0041058	42	M	2017	Pardubice	0.25	2	8	>8	0.25	≤0.125	0.008	0.125	>8	>4	1	6	27	17	t586	E	E	-	CC5	-
B0041072	77	F	2017	Prague	0.25	2	8	>8	0.5	≤0.125	0.008	0.125	>8	>4	1	6	27	16	t893	1	003	-	CC5	-
B0041098	71	F	2017	Pilsen	0.25	2	8	>8	1	≤0.125	≤0.004	0.125	>8	>4	1	6	26	20	t014	1	003	-	CC5	-
B0041127	58	M	2017	Central Bohemian	0.25	2	8	>8	0.5	0.25	0.008	0.125	>8	>4	1	6	27	18	t014	1	003	-	CC5	-
B0041173	67	F	2017	Prague	0.25	2	8	0.5	0.5	≤0.125	0.008	0.125	0.5	0.125	1	11	27	20	t304	4	024	-	CC8	-
B0041198	65	M	2017	Pilsen	0.25	2	8	>8	0.25	0.25	≤0.004	0.25	>8	>4	1	6	28	18	t003	1	003	-	CC5	-
B0041209	59	M	2017	Vysocina	0.25	2	8	>8	0.5	≤0.125	≤0.004	0.125	>8	>4	1	6	25	18	t586	E	E	-	CC5	-
B0041215	63	M	2017	Liberec	0.25	2	8	>8	0.5	≤0.125	0.008	0.125	>8	>4	1	8	28	19	t003	1	003	-	CC5	-
B0041237	71	F	2017	South Bohemian	0.25	2	8	>8	0.25	≤0.125	≤0.004	0.125	>8	>4	1	6	22	17	t586	E	E	-	CC5	-
B0041259	83	F	2017	Usti nad Labem	0.25	2	8	>8	0.5	≤0.125	≤0.004	0.125	>8	>4	1	6	26	19	t003	1	003	-	CC5	-

B0041283	83	M	2017	South Bohemian	0.25	2	8	>8	>8	0.5	>0.5	0.06	>8	>4	1	6	27	18	t586	E	E	-	CC5	-
B0041287	83	M	2017	South Bohemian	0.25	2	8	>8	0.5	0.25	0.008	0.125	0.5	0.25	1	6	26	19	t6428	1	003	-	CC5	-
B0041290	81	F	2017	South Bohemian	0.25	2	8	>8	0.5	≤0.125	0.008	0.125	>8	>4	1	6	23	17	t586	E	E	-	CC5	-
B0041291	74	M	2017	South Bohemian	0.25	2	8	>8	>8	0.25	0.008	0.125	>8	>4	1	6	8	17	t586	E	E	-	CC5	-
B0041324	67	F	2017	South Moravian	0.25	2	8	>8	0.5	≤0.125	0.008	0.125	>8	>4	1	6	26	18	t586	E	E	-	CC5	-
B0041370	83	F	2017	Hradec Kralove	0.25	2	8	>8	0.25	≤0.125	0.008	0.125	>8	**0.25**	1	6	26	18	t002	1	003	-	CC5	-
B0041377	68	M	2017	Hradec Kralove	0.25	2	8	>8	0.5	≤0.125	0.008	2	>8	**0.125**	2	6	25	18	t014	1	003	-	CC5	-
B0041426	66	M	2017	Central Bohemian	0.25	2	8	>8	0.25	≤0.125	≤0.004	0.125	>8	>4	1	6	27	23	t003	1	003	-	CC5	-
B0041437	77	M	2017	South Bohemian	0.25	2	8	>8	>8	0.25	0.008	0.125	>8	>4	1	6	26	20	t3195	1	003	-	CC5	-
B0041486	74	F	2017	Central Bohemian	0.25	2	8	>8	0.25	≤0.125	0.008	0.125	>8	**0.25**	1	6	28	19	t002	1	003	-	CC5	-
B0041503	70	F	2017	Prague	0.25	2	>64	>8	0.25	≤0.125	≤0.004	0.125	>8	>4	1	6	28	18	t586	E	E	-	CC5	-
B0041518	68	M	2017	Pardubice	0.125	2	8	>8	0.25	≤0.125	≤0.004	2	>8	**0.125**	1	6	29	19	t014	1	003	-	CC5	-
B0041539	83	M	2017	South Bohemian	0.125	2	8	>8	0.25	≤0.125	≤0.004	0.125	>8	>4	1	11	26	21	t014	1	003	-	CC5	-
B0041540	69	M	2017	South Bohemian	0.25	2	8	>8	>8	0.25	≤0.004	0.125	>8	>4	1	6	9	20	t586	E	E	-	CC5	-
B0041541	48	F	2017	South Bohemian	0.25	2	8	>8	0.5	≤0.125	≤0.004	0.125	>8	>4	1	6	26	20	t586	E	E	-	CC5	-
B0041575	74	M	2017	South Moravian	0.25	2	8	>8	0.25	≤0.125	≤0.004	0.25	>8	>4	1	6	27	20	t003	1	003	-	CC5	-
B0041594	40	M	2017	Pilsen	0.25	2	8	>8	0.5	≤0.125	0.008	0.125	>8	>4	1	6	28	20	t014	1	003	-	CC5	-
B0041612	44	M	2017	Prague	0.125	1	8	>8	>8	16	0.008	0.125	>8	>4	1	6	26	16	t041	1	003	-	CC5	-
B0041626	40	M	2017	Central Bohemian	0.25	2	8	>8	>8	1	≤0.004	0.125	>8	>4	1	6	28	20	t014	1	003	-	CC5	-
B0041668	84	M	2017	Central Bohemian	0.25	2	8	>8	0.25	≤0.125	0.008	0.125	>8	>4	1	6	27	17	t586	E	E	-	CC5	-
B0041708	70	M	2017	Liberec	0.125	2	8	>8	0.5	≤0.125	0.016	0.25	>8	>4	1	6	29	20	t586	E	E	-	CC5	-
B0041713	70	F	2017	Usti nad Labem	0.25	2	8	>8	0.25	≤0.125	0.008	0.125	>8	>4	0.5	6	30	18	t003	1	003	-	CC5	-
B0041753	53	F	2017	Prague	0.25	2	8	>8	0.25	≤0.125	0.008	0.125	>8	>4	0.5	6	26	19	t003	1	003	-	CC5	-
B0041763	70	M	2017	Hradec Kralove	0.125	1	>64	>8	>8	≤0.125	≤0.004	0.06	>8	**0.125**	1	6	31	18	t002	1	003	-	CC5	-
B0041781	83	F	2017	South Moravian	0.125	1	>64	>8	0.25	≤0.125	>0.5	0.125	>8	>4	1	6	28	17	t003	1	003	225	CC5	II
B0041792	72	M	2017	Pilsen	0.25	2	8	>8	0.5	0.25	0.008	0.125	>8	>4	1	6	25	20	t014	1	003	-	CC5	-
B0041798	79	F	2017	Olomouc	0.25	2	8	>8	0.25	≤0.125	0.008	0.125	>8	>4	0.5	6	27	18	t003	1	003	-	CC5	-
B0041805	67	M	2017	Olomouc	0.25	2	8	>8	0.25	≤0.125	0.008	0.125	>8	>4	0.5	7	28	17	t003	1	003	-	CC5	-
B0041809	72	M	2017	Central Bohemian	0.125	2	4	>8	0.25	≤0.125	0.008	0.125	>8	**0.25**	1	6	29	17	t002	1	003	-	CC5	-
B0041818	82	M	2017	Prague	0.125	2	4	0.5	0.25	≤0.125	0.008	0.125	>8	**0.125**	1	10	28	22	t127	S	S	-	CC1	-
B0041826	74	F	2017	Prague	0.25	2	8	>8	>8	0.5	0.008	0.125	>8	>4	1	6	27	19	t586	E	E	-	CC5	-
B0041828	83	F	2017	Vysocina	0.125	1	8	>8	0.5	≤0.125	≤0.004	0.125	>8	>4	0.5	13	25	22	t003	1	003	-	CC5	-
B0041829	78	F	2017	Vysocina	0.125	2	8	>8	0.25	≤0.125	0.008	0.125	>8	>4	0.5	6	27	18	t003	1	003	-	CC5	-
B0041892	83	M	2017	Prague	0.125	2	4	>8	0.25	≤0.125	0.008	0.125	0.5	0.125	0.5	14	28	22	t1282	1	003	-	CC5	-
B0041931	69	M	2017	Vysocina	0.125	2	8	>8	0.5	≤0.125	0.008	0.125	>8	>4	0.5	6	28	19	t003	1	003	-	CC5	-
B0041954	65	F	2017	Hradec Kralove	0.125	2	>64	>8	0.5	≤0.125	0.008	0.125	>8	>4	0.5	6	28	21	t014	1	003	-	CC5	-
B0041969	85	F	2017	South Moravian	0.125	2	8	>8	0.25	0.5	≤0.004	0.125	>8	>4	0.5	6	27	18	t003	1	003	-	CC5	-
B0042027	86	M	2017	Vysocina	0.125	2	8	>8	0.25	≤0.125	0.008	0.125	>8	>4	0.5	6	28	20	t003	1	003	-	CC5	-

B0042044	70	M	2017	Vysocina	0.125	2	8	>8	0.25	≤0.125	0.008	0.25	>8	>4	0.5	6	27	19	t003	1	003	-	CC5	-
B0042094	80	F	2017	Olomouc	0.125	1	8	>8	0.25	≤0.125	≤0.004	0.125	>8	>4	0.5	6	27	19	t003	1	003	-	CC5	-
B0042118	68	M	2017	Prague	0.125	2	4	0.5	0.25	≤0.125	0.008	0.125	>8	**0.125**	1	6	9	20	t127	S	S	1	CC1	IV
B0042119	61	M	2017	Prague	0.125	2	8	>8	>8	≤0.125	≤0.004	0.125	>8	>4	0.5	6	11	21	t014	1	003	-	CC5	-
B0042160	68	M	2017	South Bohemian	0.125	2	8	>8	>8	1	>0.5	0.25	>8	>4	1	6	27	19	t586	E	E	-	CC5	-
B0042161	85	F	2017	South Bohemian	0.25	2	8	>8	>8	0.25	≤0.004	0.125	>8	>4	1	6	28	18	t586	E	E	-	CC5	-
B0042163	74	F	2017	South Bohemian	0.125	2	8	>8	>8	0.25	≤0.004	0.125	>8	>4	1	6	29	19	t586	E	E	-	CC5	-
B0042170	79	F	2017	South Bohemian	0.25	2	8	>8	>8	0.25	≤0.004	0.125	>8	>4	1	6	8	19	t586	E	E	-	CC5	-
B0042176	34	F	2017	South Bohemian	0.125	2	8	>8	0.25	≤0.125	0.008	0.125	>8	>4	1	6	26	20	t003	1	003	-	CC5	-
B0042177	70	M	2017	South Bohemian	0.25	2	8	>8	0.25	≤0.125	≤0.004	0.125	>8	>4	0.5	6	28	18	t003	1	003	-	CC5	-
B0042199	62	F	2017	Prague	0.125	2	4	2	0.5	≤0.125	≤0.004	0.125	>8	**0.125**	0.5	15	10	24	t127	S	S	-	CC1	-
B0042270	86	F	2017	Vysocina	0.25	2	8	>8	0.5	≤0.125	0.008	0.125	>8	>4	0.5	9	24	19	t003	1	003	-	CC5	-
B0042284	83	F	2017	Hradec Kralove	0.125	1	8	>8	0.25	≤0.125	0.008	0.125	>8	>4	0.5	6	27	19	t003	1	003	-	CC5	-
B0042289	86	F	2017	Prague	0.25	2	8	>8	0.125	≤0.125	0.008	0.125	>8	>4	0.5	6	25	21	t151	1	003	-	CC5	-
B0042321	68	M	2017	Pilsen	0.125	2	8	>8	0.25	≤0.125	0.008	0.125	>8	>4	0.5	6	27	19	t003	1	003	-	CC5	-
B0042384	69	F	2017	Vysocina	0.125	2	8	>8	0.25	≤0.125	≤0.004	0.125	>8	**0.25**	0.5	6	24	19	t002	1	003	5	CC5	II
B0042411	66	M	2018	Olomouc	0.25	2	8	>8	0.25	≤0.125	0.008	0.125	>8	>4	0.5	7	27	19	t003	1	003	-	CC5	-
B0042484	72	F	2017	Karlovy Vary	0.125	1	8	>8	1	≤0.125	>0.5	0.06	>8	>4	1	6	32	18	t014	1	003	-	CC5	-
B0042509	53	M	2017	Karlovy Vary	0.125	2	8	>8	0.25	≤0.125	≤0.004	0.125	>8	>4	0.5	6	28	20	t014	1	003	-	CC5	-
B0042602	77	M	2018	Pardubice	0.25	2	8	>8	0.5	0.25	0.008	0.125	>8	>4	1	6	30	20	t014	1	003	-	CC5	-
B0042606	64	M	2017	South Bohemian	0.25	2	8	>8	0.25	≤0.125	0.008	0.25	>8	>4	0.5	6	29	18	t586	E	E	-	CC5	-
B0042608	72	M	2017	South Bohemian	0.25	2	8	>8	>8	0.5	0.016	0.125	>8	>4	1	6	9	17	t586	E	E	-	CC5	-
B0042633	73	M	2018	Pilsen	0.25	2	8	>8	0.25	≤0.125	0.008	0.125	>8	>4	0.5	6	29	23	t003	1	003	-	CC5	-
B0042647	62	M	2017	Prague	0.125	2	8	>8	0.25	≤0.125	0.008	0.125	>8	**0.25**	0.5	6	27	17	t586	E	E	-	CC5	-
B0042704	47	M	2018	Usti nad Labem	0.25	4	8	>8	0.5	≤0.125	0.008	0.25	>8	>4	0.5	6	27	18	t003	1	003	-	CC5	-
B0042705	80	F	2018	Usti nad Labem	0.25	2	8	>8	0.5	≤0.125	0.008	0.125	>8	>4	1	6	26	18	t003	1	003	-	CC5	-
B0042717	48	M	2018	Prague	0.125	2	8	>8	>8	≤0.125	0.06	4	>8	>4	0.5	6	13	18	t003	1	003	-	CC5	-
B0042732	71	M	2018	South Bohemian	0.125	1	4	>8	0.5	≤0.125	≤0.004	0.125	>8	>4	0.5	6	30	19	t003	1	003	-	CC5	-
B0042736	46	M	2018	Hradec Kralove	0.125	2	64	>8	>8	≤0.125	≤0.004	0.125	>8	>4	0.5	8	29	17	t1282	1	003	-	CC5	-
B0042775	73	F	2018	Vysocina	0.25	2	8	>8	0.25	≤0.125	0.008	0.125	>8	>4	0.5	6	31	19	t003	1	003	-	CC5	-
B0042783	77	M	2018	South Bohemian	0.125	2	8	>8	0.25	≤0.125	0.008	0.125	>8	>4	0.5	6	30	19	t586	E	E	-	CC5	-
B0042803	66	F	2018	Pardubice	0.125	2	8	>8	0.5	0.25	0.008	0.125	>8	>4	1	6	31	21	t014	1	003	-	CC5	-
B0042834	71	M	2018	South Moravian	0.125	1	8	>8	0.25	≤0.125	0.008	0.125	>8	>4	1	6	33	19	t003	1	003	-	CC5	-
B0042934	71	M	2018	Central Bohemian	0.125	1	4	>8	0.125	≤0.125	≤0.004	0.125	>8	>4	0.5	6	29	18	t003	1	003	-	CC5	-
B0042966	60	M	2018	Pardubice	0.125	2	8	>8	0.25	16	0.008	0.125	>8	>4	0.5	6	14	15	t014	1	003	-	CC5	-
B0042983	64	M	2018	Hradec Kralove	0.125	2	8	>8	0.25	≤0.125	≤0.004	0.25	>8	>4	0.5	6	26	19	t014	1	003	-	CC5	-
B0042987	88	M	2018	Prague	0.125	2	8	>8	0.5	≤0.125	0.008	0.125	>8	>4	0.5	6	28	18	t003	1	003	-	CC5	-
B0043059	67	M	2018	Usti nad Labem	0.125	1	8	>8	0.25	≤0.125	≤0.004	0.06	>8	>4	0.5	7	29	18	t003	1	003	-	CC5	-
B0043071	75	M	2018	Vysocina	0.25	2	8	>8	0.25	≤0.125	0.008	0.25	>8	>4	0.5	6	28	18	t586	E	E	-	CC5	-

B0043091	61	M	2018	Pilsen	0.25	2	8	>8	0.25	≤0.125	0.008	0.25	>8	>4	0.5	6	29	14	t003	1	003	-	CC5	-
B0043093	69	M	2018	Pilsen	0.25	2	8	>8	0.25	≤0.125	0.008	0.25	>8	>4	1	14	30	19	t014	1	003	-	CC5	-
B0043133	72	F	2018	Prague	0.125	2	>64	>8	0.5	≤0.125	≤0.004	0.125	>8	>4	0.5	6	28	19	t586	E	E	-	CC5	-
B0043144	66	M	2018	Central Bohemian	0.25	2	4	>8	0.5	≤0.125	0.008	>4	>8	**0.25**	1	6	27	18	t002	1	003	-	CC5	-
B0043151	89	F	2018	South Bohemian	0.25	2	8	>8	0.5	≤0.125	0.008	0.125	>8	>4	0.5	6	24	19	t003	1	003	-	CC5	-
B0043165	82	M	2018	South Bohemian	0.25	2	8	>8	>8	0.25	≤0.004	0.125	>8	>4	0.5	6	11	18	t586	E	E	225	CC5	II
B0043167	81	F	2018	South Bohemian	0.25	1	8	>8	>8	0.25	≤0.004	0.06	>8	>4	0.5	6	12	19	t586	E	E	-	CC5	-
B0043199	60	M	2018	Pilsen	0.25	2	8	>8	0.25	≤0.125	0.008	0.25	>8	**0.25**	1	6	27	20	t003	1	003	-	CC5	-
B0043212	84	M	2018	Vysocina	0.25	2	8	>8	0.5	0.5	≤0.004	0.125	0.5	>4	0.5	19	7	23	t034	2	011	398	CC398	V
B0043218	62	M	2018	Prague	0.125	2	8	>8	0.25	≤0.125	≤0.004	0.125	>8	>4	0.5	14	29	21	t003	1	003	-	CC5	-
B0043319	74	M	2018	Olomouc	0.125	2	8	>8	0.25	≤0.125	≤0.004	0.125	>8	>4	0.5	11	27	19	t003	1	003	-	CC5	-
B0043465	58	M	2018	Central Bohemian	0.25	2	8	>8	0.5	≤0.125	0.008	0.25	>8	>4	1	8	28	19	t014	1	003	-	CC5	-
B0043489	84	F	2018	Prague	0.25	2	16	>8	0.5	≤0.125	0.008	0.25	>8	>4	1	6	27	18	t586	E	E	-	CC5	-
B0043541	66	M	2018	Prague	0.125	2	4	0.5	0.5	≤0.125	0.008	0.125	>8	>4	1	13	10	21	t127	S	S	-	CC1	-
B0043542	79	M	2018	Prague	0.25	2	8	>8	0.5	≤0.125	0.008	0.25	>8	>4	1	6	26	17	t586	E	E	-	CC5	-
B0043557	62	M	2018	Zlin	0.25	2	8	>8	0.5	0.25	0.008	0.25	>8	>4	1	6	28	17	t003	1	003	-	CC5	-
B0043559	80	F	2018	Zlin	0.25	2	8	>8	0.5	0.25	0.008	0.125	>8	>4	1	8	27	19	t003	1	003	-	CC5	-
B0043561	73	M	2018	Zlin	0.25	2	8	>8	0.5	0.25	≤0.004	0.125	>8	>4	1	6	29	17	t003	1	003	-	CC5	-
B0043585	74	F	2018	Usti nad Labem	0.25	2	8	>8	0.5	≤0.125	0.008	0.125	>8	>4	0.5	11	30	18	t003	1	003	-	CC5	-
B0043602	92	F	2018	Prague	0.25	2	8	>8	0.5	≤0.125	0.008	0.125	>8	>4	0.5	6	31	20	t3560	1	003	-	CC5	-
B0043638	65	M	2018	Central Bohemian	0.25	2	8	>8	0.5	≤0.125	0.008	0.125	>8	**0.25**	0.5	7	27	19	t1227	1	003	-	CC5	-
B0043640	66	M	2018	Liberec	0.25	2	>64	>8	>8	≤0.125	≤0.004	0.06	>8	**0.125**	0.5	9	28	17	t002	1	003	-	CC5	-
B0043643	73	M	2018	South Moravian	0.125	2	4	>8	0.5	≤0.125	≤0.004	0.125	>8	**0.125**	0.5	8	28	23	t18037	1	003	-	CC5	-
B0043674	9	F	2018	Prague	0.25	2	8	>8	0.5	≤0.125	0.008	0.125	>8	0.125	1	15	30	19	t008	4	024	8	CC8	IV
B0043710	64	F	2018	Prague	0.125	1	8	>8	0.5	>16	0.008	>4	>8	>4	2	6	11	18	t003	1	003	-	CC5	-
B0043731	62	M	2018	Central Bohemian	0.25	2	8	>8	0.25	≤0.125	0.008	4	>8	**0.25**	1	6	30	17	t002	1	003	-	CC5	-
B0043746	73	M	2018	Hradec Kralove	0.125	1	4	0.5	0.5	0.25	0.008	0.06	0.25	0.125	1	12	25	21	t015	5	NF	45	CC45	IV
B0043756	82	F	2018	Prague	0.125	1	8	>8	0.25	≤0.125	≤0.004	0.25	>8	>4	0.5	6	30	18	t586	E	E	-	CC5	-
B0043761	80	M	2018	Prague	0.25	2	8	>8	0.5	0.25	0.008	0.125	>8	>4	0.5	8	29	19	t003	1	003	-	CC5	-
B0043782	62	M	2018	Pardubice	0.125	2	4	>8	0.5	≤0.125	≤0.004	0.125	>8	**0.25**	0.5	6	29	19	t002	1	003	-	CC5	-
B0043791	70	F	2018	South Moravian	0.125	1	8	>8	0.25	≤0.125	0.008	0.125	>8	>4	0.5	6	29	19	t003	1	003	-	CC5	-
B0043803	63	M	2018	Prague	0.25	2	8	>8	0.5	>16	≤0.004	0.25	>8	>4	1	6	31	19	t003	1	003	-	CC5	-
B0043814	67	F	2018	Pardubice	0.25	2	8	>8	0.5	0.25	≤0.004	0.125	>8	>4	1	6	30	19	t003	1	003	-	CC5	-
B0043844	60	M	2018	Prague	0.25	2	8	>8	0.25	≤0.125	>0.5	0.25	>8	>4	1	6	30	18	t586	E	E	-	CC5	-
B0043848	38	M	2018	Prague	0.25	2	8	>8	0.5	0.25	0.008	0.125	>8	>4	1	13	29	20	t008	4	024	8	CC8	IV
B0043855	38	M	2018	Vysocina	0.125	2	8	>8	0.5	≤0.125	0.008	0.125	>8	>4	1	6	29	19	t014	1	003	-	CC5	-
B0043901	81	M	2018	Usti nad Labem	0.25	2	8	>8	0.5	≤0.125	0.008	0.125	>8	>4	0.5	6	30	18	t003	1	003	-	CC5	-
B0043904	64	F	2018	Usti nad Labem	0.25	2	8	>8	1	≤0.125	0.008	0.125	>8	>4	0.5	6	29	19	t003	1	003	-	CC5	-
B0043907	62	M	2018	Usti nad Labem	0.125	1	8	>8	0.5	≤0.125	≤0.004	0.125	>8	>4	0.5	15	29	22	t003	1	003	-	CC5	-
B0043909	65	F	2018	Usti nad Labem	0.25	2	8	>8	0.5	≤0.125	0.008	0.125	>8	>4	0.5	7	28	20	t003	1	003	-	CC5	-

B0043952	74	F	2018	Prague	0.25	2	8	>8	0.25	≤0.125	0.008	0.125	>8	>4	0.5	6	30	19	t003	1	003	-	CC5	-
B0043954	76	F	2018	Prague	0.125	1	4	>8	0.25	≤0.125	>0.5	0.125	>8	**0.125**	0.5	6	30	18	t003	1	003	-	CC5	-
B0043980	86	F	2018	Karlovy Vary	0.125	2	8	>8	0.25	≤0.125	0.008	0.125	>8	>4	0.5	6	30	20	t003	1	003	-	CC5	-
B0043999	71	M	2018	Karlovy Vary	0.25	2	8	>8	0.5	≤0.125	≤0.004	0.125	>8	>4	0.5	6	28	17	t586	E	E	-	CC5	-
B0044001	88	M	2018	Karlovy Vary	0.125	1	8	>8	0.25	0.25	≤0.004	0.125	>8	>4	0.5	6	28	18	t003	1	003	-	CC5	-
B0044015	89	M	2018	Pilsen	0.25	2	8	>8	0.5	≤0.125	≤0.004	0.125	>8	>4	0.5	6	28	21	t014	1	003	-	CC5	-
B0044019	34	M	2018	Pilsen	0.125	2	8	1	0.5	≤0.125	0.008	0.125	0.5	0.125	0.5	13	12	22	t437	S	S	-	CC59	-
B0044021	71	M	2018	South Bohemian	0.125	1	4	>8	>8	2	0.008	0.25	>8	>4	0.5	6	29	17	t586	E	E	-	CC5	-
B0044022	64	M	2018	South Bohemian	0.125	2	8	>8	0.25	≤0.125	>0.5	0.125	>8	>4	1	6	28	19	t586	E	E	-	CC5	-
B0044038	45	F	2018	Central Bohemian	0.25	2	8	>8	0.5	0.25	0.008	2	>8	**0.25**	1	6	26	20	t002	1	003	-	CC5	-
B0044043	70	M	2018	South Bohemian	0.125	1	8	>8	>8	0.25	≤0.004	0.125	>8	>4	0.5	6	30	21	t586	E	E	-	CC5	-
B0044049	79	M	2018	South Bohemian	0.125	1	4	>8	0.25	≤0.125	≤0.004	0.125	>8	**0.125**	0.5	10	33	20	t014	1	003	-	CC5	-
B0044051	62	M	2018	Prague	0.125	2	8	>8	0.25	≤0.125	0.008	0.125	>8	>4	0.5	6	32	25	t264	1	003	-	CC5	-
B0044088	59	M	2018	Pardubice	0.25	2	8	>8	0.25	≤0.125	0.008	0.125	>8	>4	0.5	9	29	20	t014	1	003	-	CC5	-
B0044112	50	M	2018	Pardubice	0.25	2	8	>8	0.5	0.25	0.008	0.5	>8	>4	0.5	11	29	19	t014	1	003	-	CC5	-
B0044114	76	F	2018	South Bohemian	0.25	2	8	>8	0.25	0.5	0.008	0.25	>8	>4	1	6	31	19	t003	1	003	-	CC5	-
B0044135	66	F	2018	South Bohemian	0.25	2	8	>8	0.5	≤0.125	0.008	0.25	>8	>4	0.5	6	31	18	t586	E	E	-	CC5	-
B0044162	84	F	2018	South Bohemian	0.25	2	8	>8	0.5	≤0.125	0.008	0.25	>8	>4	0.5	7	10	20	t003	1	003	-	CC5	-
B0044173	71	M	2018	Prague	0.125	1	8	>8	0.5	0.5	≤0.004	0.125	>8	>4	0.5	10	34	22	t045	1	003	-	CC5	-
B0044221	79	M	2018	Usti nad Labem	0.25	2	8	>8	0.25	≤0.125	0.008	0.25	>8	>4	0.5	7	25	21	t003	1	003	-	CC5	-
B0044232	74	M	2018	Prague	0.25	2	8	>8	0.5	0.25	0.008	0.25	>8	>4	0.5	6	26	19	t003	1	003	-	CC5	-
B0044249	69	M	2018	Prague	0.125	2	16	>8	0.5	0.25	0.008	0.125	>8	>4	1	6	26	20	t003	1	003	-	CC5	-
B0044257	70	F	2018	Liberec	0.125	2	8	>8	0.5	≤0.125	≤0.004	0.125	>8	>4	0.5	10	27	20	t586	E	E	-	CC5	-
B0044299	68	F	2018	Usti nad Labem	0.25	2	8	>8	0.5	≤0.125	0.008	0.25	>8	>4	0.5	6	25	20	t003	1	003	-	CC5	-
B0044317	45	F	2018	Prague	0.25	2	8	>8	0.5	0.25	0.008	>4	>8	**0.25**	1	6	27	17	t002	1	003	-	CC5	-
B0044406	56	F	2018	Olomouc	0.25	2	8	>8	0.5	≤0.125	0.008	0.125	>8	>4	0.5	10	29	21	t003	1	003	-	CC5	-
B0044410	71	F	2018	Olomouc	0.25	2	8	>8	0.5	≤0.125	0.008	0.125	>8	>4	0.5	6	28	20	t003	1	003	-	CC5	-
B0044414	58	F	2018	Prague	0.125	1	8	>8	>8	0.5	≤0.004	0.125	>8	>4	1	6	27	19	t586	E	E	-	CC5	-
B0044424	89	F	2018	South Moravian	0.25	2	8	>8	0.5	≤0.125	0.008	0.125	>8	>4	1	6	29	22	t003	1	003	-	CC5	-
B0044428	87	F	2018	Vysocina	0.25	2	8	>8	0.5	≤0.125	0.008	0.125	>8	>4	0.5	10	27	23	t014	1	003	-	CC5	-
B0044462	60	M	2018	South Bohemian	0.125	2	8	0.125	0.25	≤0.125	0.008	0.25	0.5	0.25	1	13	25	22	t1231	5	NF	45	CC45	IV
B0044470	60	F	2018	South Moravian	0.5	2	8	0.5	0.25	1	0.008	0.125	>8	>4	0.5	16	6	23	t034	2	011	-	CC398	-
B0044508	44	F	2018	South Bohemian	0.125	2	8	>8	0.25	≤0.125	0.008	0.5	>8	>4	1	6	28	19	t586	E	E	-	CC5	-
B0044509	65	F	2018	South Bohemian	0.25	2	16	>8	0.5	≤0.125	0.008	0.25	>8	>4	1	6	23	20	t586	E	E	-	CC5	-
B0044559	56	M	2018	Pilsen	0.25	2	8	>8	0.5	≤0.125	0.008	0.125	>8	>4	0.5	6	26	18	t014	1	003	-	CC5	-
B0044569	56	M	2018	Hradec Kralove	0.25	2	8	>8	0.5	≤0.125	0.008	0.25	>8	>4	0.5	8	24	19	t003	1	003	-	CC5	-
B0044584	76	F	2018	Prague	0.25	2	8	0.5	0.5	2	0.008	0.125	>8	>4	0.5	15	6	20	t3275	2	011	-	CC398	-
B0044592	59	M	2018	Pilsen	0.25	2	8	>8	0.25	≤0.125	0.008	0.125	>8	>4	1	6	26	19	t003	1	003	-	CC5	-
B0044602	73	M	2018	Prague	0.125	1	8	>8	0.25	≤0.125	0.008	0.125	>8	>4	0.5	6	28	22	t003	1	003	-	CC5	-
B0044613	50	M	2018	Prague	0.25	2	8	>8	0.5	≤0.125	0.008	0.25	>8	>4	1	6	27	20	t586	E	E	-	CC5	-

B0044663	76	M	2018	Pardubice	0.25	2	16	>8	0.5	0.25	0.008	0.25	>8	>4	0.5	6	30	21	t003	1	003	-	CC5	-
B0044705	69	F	2018	Prague	0.125	2	8	>8	0.25	0.5	0.016	0.25	>8	>4	1	6	26	19	t586	E	E	-	CC5	-
B0044732	87	F	2018	Karlovy Vary	0.125	2	8	>8	0.5	≤0.125	0.008	0.125	>8	>4	0.5	6	29	19	t003	1	003	-	CC5	-
B0044733	81	F	2018	Karlovy Vary	0.25	2	8	>8	0.5	0.25	0.008	0.25	>8	**0.25**	0.5	6	26	17	t003	1	003	-	CC5	-
B0044843	75	M	2018	South Moravian	0.25	2	8	0.5	0.25	0.5	≤0.004	0.06	>8	>4	1	16	8	21	t034	2	011	398	CC398	V
B0044846	91	M	2018	Hradec Kralove	0.125	2	8	>8	0.5	≤0.125	0.008	0.125	>8	>4	1	6	26	20	t014	1	003	-	CC5	-
B0044857	80	F	2018	Vysocina	0.25	2	8	>8	0.25	≤0.125	0.008	0.125	>8	**0.125**	1	6	27	20	t014	1	003	-	CC5	-
B0044884	60	M	2018	Prague	0.25	2	8	1	1	≤0.125	0.008	0.125	>8	**0.25**	1	12	30	20	t010	1	003	-	CC5	-
B0044903	85	M	2018	South Moravian	0.125	2	8	>8	0.25	≤0.125	0.008	0.25	>8	>4	1	10	30	19	t003	1	003	-	CC5	-
B0044949	78	F	2018	South Moravian	0.25	2	8	>8	0.5	≤0.125	0.008	0.25	>8	>4	1	6	30	21	t003	1	003	-	CC5	-
B0044950	79	M	2018	South Moravian	0.25	2	8	>8	0.25	≤0.125	0.008	0.25	>8	>4	1	6	30	21	t003	1	003	-	CC5	-
B0045007	76	M	2018	Hradec Kralove	0.5	2	8	1	0.5	4	0.008	0.25	>8	>4	0.5	14	6	20	t588	2	011	-	CC398	-
B0045015	66	F	2018	Central Bohemian	0.125	2	8	>8	0.25	≤0.125	≤0.004	0.125	>8	>4	0.5	13	25	25	t586	E	E	-	CC5	-
B0045078	66	M	2018	Prague	0.25	2	8	>8	0.5	≤0.125	0.008	0.25	>8	>4	1	6	27	19	t003	1	003	-	CC5	-
B0045098	60	M	2018	Prague	0.25	4	8	1	0.5	0.25	>0.5	0.125	>8	**0.25**	2	14	32	21	t010	1	003	-	CC5	-
B0045137	83	F	2018	Central Bohemian	0.25	2	8	>8	0.5	≤0.125	0.008	0.125	>8	**0.25**	1	6	28	19	t002	1	003	-	CC5	-
B0045220	59	M	2018	Pilsen	0.25	2	16	>8	0.5	≤0.125	0.008	0.25	>8	>4	0.5	6	29	18	t003	1	003	-	CC5	-
B0045251	64	M	2018	Usti nad Labem	0.25	2	16	>8	0.5	≤0.125	0.008	0.25	>8	>4	1	15	33	24	t003	1	003	-	CC5	-
B0045310	84	F	2018	Prague	0.25	2	8	>8	0.25	≤0.125	0.008	0.125	>8	>4	1	6	27	18	t586	E	E	-	CC5	-
B0045322	52	M	2018	Prague	0.125	2	8	>8	0.5	≤0.125	≤0.004	0.125	>8	>4	1	6	29	18	t586	E	E	-	CC5	-
B0045326	83	F	2018	Prague	0.25	2	8	>8	0.5	≤0.125	0.008	0.125	>8	>4	0.5	6	28	18	t586	E	E	-	CC5	-
B0045336	0	F	2018	Vysocina	0.25	2	8	>8	0.5	≤0.125	≤0.004	0.125	>8	>4	1	6	29	19	t586	E	E	-	CC5	-
B0045342	0	F	2018	Prague	0.25	2	8	>8	0.5	≤0.125	0.016	0.25	>8	>4	1	6	28	18	t586	E	E	-	CC5	-
B0045349	0	F	2018	Prague	0.25	2	8	>8	0.5	≤0.125	0.008	0.125	>8	>4	1	6	26	17	t586	E	E	-	CC5	-
B0045443	80	F	2018	Prague	0.25	2	8	>8	0.5	≤0.125	≤0.004	0.125	>8	>4	1	6	27	18	t586	E	E	-	CC5	-
B0045477	76	F	2018	Pilsen	0.125	2	8	>8	0.25	≤0.125	0.008	0.125	>8	>4	0.5	6	29	18	t003	1	003	-	CC5	-
B0045480	70	M	2018	Pilsen	0.25	2	8	>8	0.25	≤0.125	0.008	0.25	>8	>4	0.5	6	29	18	t003	1	003	-	CC5	-
B0045519	89	M	2018	Pardubice	0.125	1	4	>8	0.25	≤0.125	0.008	0.125	>8	**0.125**	1	6	34	21	t014	1	003	-	CC5	-
B0045541	85	F	2018	Prague	0.25	2	16	>8	>8	0.5	0.008	0.125	>8	>4	1	6	29	20	t586	E	E	-	CC5	-
B0045550	79	M	2018	Usti nad Labem	0.125	2	8	0.5	>8	≤0.125	≤0.004	>4	0.5	0.125	1	16	28	21	t4000	S	S	72	CC8	nt
B0045585	69	M	2018	South Bohemian	0.125	2	8	>8	>8	0.25	≤0.004	0.25	>8	>4	0.5	9	11	19	t586	E	E	-	CC5	-
B0045693	86	M	2018	Pilsen	0.25	4	16	>8	0.5	0.25	0.008	0.25	>8	>4	0.5	9	28	21	t014	1	003	-	CC5	-
B0045843	86	F	2018	South Bohemian	0.25	2	8	>8	>8	0.25	≤0.004	0.125	>8	>4	0.5	6	8	18	t586	E	E	-	CC5	-
B0045856	33	F	2018	Prague	0.25	2	8	>8	0.5	≤0.125	0.008	0.125	>8	>4	1	6	32	18	t003	1	003	-	CC5	-
B0045883	59	M	2018	South Moravian	0.125	2	8	>8	0.5	≤0.125	0.008	0.25	>8	>4	1	9	30	19	t003	1	003	-	CC5	-
B0045915	54	M	2018	Pilsen	0.5	2	8	>8	0.5	0.25	≤0.004	0.25	>8	>4	0.5	6	31	19	t003	1	003	-	CC5	-
B0045953	63	M	2018	Karlovy Vary	0.25	2	8	>8	0.5	≤0.125	0.008	0.25	>8	>4	0.5	6	25	19	t586	E	E	-	CC5	-
B0045958	70	M	2018	Karlovy Vary	0.125	2	8	>8	0.25	≤0.125	≤0.004	0.125	>8	>4	0.5	6	27	17	t003	1	003	-	CC5	-
B0045983	39	M	2018	Pilsen	0.25	2	8	>8	0.25	≤0.125	0.008	0.25	>8	>4	0.5	10	25	19	t014	1	003	-	CC5	-
B0045994	79	F	2018	South Moravian	0.25	2	8	>8	0.5	0.25	0.008	0.25	>8	>4	1	6	30	20	t003	1	003	-	CC5	-

B0046007	51	M	2018	Prague	0.25	2	8	>8	>8	>16	0.008	0.125	0.5	0.25	1	12	10	20	t011	2	011	398	CC398	IV
B0046028	63	M	2018	Prague	0.125	2	8	>8	0.5	≤0.125	≤0.004	0.125	>8	>4	0.5	14	26	22	t003	1	003	-	CC5	-
B0046149	36	F	2018	Central Bohemian	0.25	2	8	1	>8	≤0.125	0.008	0.125	>8	>4	1	15	23	20	t437	S	S	-	CC59	-
B0046179	85	F	2018	Central Bohemian	0.25	2	8	>8	0.5	≤0.125	0.008	0.25	>8	>4	1	6	29	17	t014	1	003	-	CC5	-
B0046187	63	M	2018	Central Bohemian	0.25	2	8	>8	0.5	≤0.125	0.008	0.125	>8	**0.25**	1	6	28	18	t002	1	003	-	CC5	-
B0046244	67	M	2018	Olomouc	0.125	2	8	>8	0.5	≤0.125	≤0.004	0.125	>8	>4	0.5	12	30	19	t003	1	003	-	CC5	-
B0046249	71	M	2018	Central Bohemian	0.25	2	8	>8	0.5	≤0.125	≤0.004	0.25	>8	**0.25**	1	7	26	19	t586	E	E	-	CC5	-
B0046250	81	M	2018	Central Bohemian	0.125	2	8	>8	0.5	0.25	≤0.004	0.125	>8	>4	0.5	14	28	23	t003	1	003	-	CC5	-
B0046253	83	M	2018	Prague	0.25	2	8	>8	0.5	>16	0.008	0.125	>8	>4	1	6	10	19	t003	1	003	-	CC5	-
B0046333	71	M	2018	Hradec Kralove	0.25	2	8	>8	0.25	≤0.125	0.008	0.125	>8	>4	1	6	30	18	t014	1	003	-	CC5	-
B0046342	69	M	2018	Prague	0.125	2	8	>8	0.5	≤0.125	≤0.004	0.125	>8	>4	1	10	26	18	t014	1	003	-	CC5	-
B0046369	74	M	2018	Prague	0.125	0.5	4	>8	0.5	≤0.125	≤0.004	≤0.03	>8	4	1	10	29	20	t003	1	003	-	CC5	-
B0046370	65	M	2018	Prague	0.25	2	8	>8	0.5	≤0.125	0.008	0.25	>8	>4	1	10	26	25	t003	1	003	-	CC5	-
B0046427	58	F	2018	Prague	0.25	2	8	>8	>8	≤0.125	0.008	>4	0.5	0.25	1	14	26	22	t008	4	024	-	CC8	-
B0046428	77	F	2018	Prague	0.125	1	8	>8	0.25	≤0.125	≤0.004	0.06	>8	>4	0.5	12	26	19	t003	1	003	-	CC5	-
B0046461	16	M	2018	Prague	0.25	2	8	>8	>8	1	0.008	0.25	>8	>4	0.5	9	26	20	t014	1	003	-	CC5	-
B0046495	87	M	2018	Vysocina	0.125	2	8	>8	0.5	≤0.125	≤0.004	0.125	>8	>4	1	10	28	18	t003	1	003	-	CC5	-
B0046501	77	F	2018	Prague	0.06	0.5	4	>8	0.25	≤0.125	≤0.004	≤0.03	>8	>4	0.5	14	6	23	t003	1	003	-	CC5	-
B0046529	72	M	2018	Pardubice	0.25	2	8	>8	0.5	≤0.125	0.008	0.125	>8	**0.25**	1	8	23	18	t18037	1	003	-	CC5	-
B0046563	48	F	2018	South Bohemian	0.125	2	8	>8	0.25	≤0.125	0.008	0.125	>8	>4	0.5	6	26	16	t586	E	E	-	CC5	-
B0046574	40	M	2018	South Bohemian	0.25	2	8	>8	0.25	≤0.125	0.008	0.125	>8	>4	1	6	29	19	t586	E	E	-	CC5	-
B0046579	64	M	2018	South Bohemian	0.25	2	8	>8	0.25	≤0.125	0.008	0.125	>8	>4	1	6	26	17	t586	E	E	-	CC5	-
B0046580	63	M	2018	South Bohemian	0.25	2	8	>8	>8	0.25	≤0.004	0.125	>8	>4	0.5	6	9	18	t586	E	E	-	CC5	-
B0046723	68	M	2018	Central Bohemian	0.125	2	8	>8	>8	0.25	≤0.004	0.125	>8	>4	1	6	29	19	t002	1	003	-	CC5	-
B0046743	84	M	2018	Pardubice	0.25	2	8	>8	1	0.25	0.03	0.25	>8	**0.25**	0.5	8	28	20	t014	1	003	-	CC5	-
B0046773	80	F	2018	Zlin	0.125	1	4	>8	1	≤0.125	0.008	0.5	>8	>4	1	12	30	22	t003	1	003	-	CC5	-
B0046781	63	M	2018	South Moravian	0.125	1	8	>8	0.5	≤0.125	≤0.004	≤0.03	>8	>4	1	9	27	18	t003	1	003	-	CC5	-
B0046794	29	M	2018	Prague	0.25	2	64	>8	1	≤0.125	≤0.004	0.125	>8	>4	1	6	28	18	t586	E	E	-	CC5	-
B0046816	86	F	2018	Pardubice	0.25	2	16	>8	0.5	≤0.125	0.008	0.125	>8	>4	1	6	25	20	t003	1	003	-	CC5	-
B0046839	65	M	2018	Central Bohemian	0.25	2	8	>8	0.5	≤0.125	0.008	0.25	>8	>4	1	7	26	18	t586	E	E	-	CC5	-
B0046862	93	F	2018	Prague	0.125	1	8	>8	0.5	≤0.125	≤0.004	0.06	>8	>4	1	8	27	19	t014	1	003	-	CC5	-
B0046924	66	F	2018	Usti nad Labem	0.25	2	8	>8	0.5	≤0.125	0.008	0.125	>8	>4	1	6	25	18	t003	1	003	-	CC5	-
B0047018	71	M	2018	Prague	0.125	1	8	>8	0.25	≤0.125	≤0.004	0.125	>8	>4	1	7	28	18	t003	1	003	-	CC5	-
B0047063	50	F	2018	Pilsen	0.25	2	8	>8	0.5	≤0.125	0.008	0.25	>8	>4	1	6	28	18	t003	1	003	225	CC5	II
B0047075	68	M	2018	South Bohemian	0.125	2	>64	>8	0.5	≤0.125	≤0.004	0.125	>8	>4	1	12	26	20	t586	E	E	-	CC5	-
B0047090	78	M	2018	Prague	0.25	2	8	>8	0.5	≤0.125	0.008	0.25	>8	>4	1	6	27	17	t586	E	E	-	CC5	-
B0047127	51	M	2018	Central Bohemian	0.25	2	8	>8	0.25	≤0.125	0.008	0.125	>8	**0.25**	1	6	27	16	t002	1	003	-	CC5	-
B0047151	75	F	2018	Pardubice	0.125	2	8	>8	0.5	≤0.125	0.008	0.125	>8	>4	0.5	9	25	18	t014	1	003	-	CC5	-
B0047166	66	M	2018	South Moravian	0.125	2	8	>8	0.5	0.25	0.008	0.125	>8	>4	1	9	27	19	t003	1	003	-	CC5	-
B0047175	74	F	2018	Pilsen	0.25	2	8	>8	0.5	0.25	0.008	0.25	>8	>4	1	9	27	19	t003	1	003	-	CC5	-

B0047178	74	M	2018	Pilsen	0.25	2	16	>8	0.5	≤0.125	0.008	1	>8	>4	1	6	25	20	t014	1	003	-	CC5	-
B0047191	82	M	2018	South Bohemian	0.25	2	8	>8	0.25	≤0.125	≤0.004	0.125	>8	>4	1	6	26	18	t586	E	E	-	CC5	-
B0047229	50	M	2018	Prague	0.5	2	8	>8	2	≤0.125	≤0.004	0.25	>8	>4	0.5	8	30	19	t003	1	003	-	CC5	-
B0047230	77	M	2018	Prague	0.25	2	8	>8	0.25	≤0.125	≤0.004	0.125	>8	>4	0.5	6	24	19	t586	E	E	-	CC5	-
B0047247	0	M	2018	Prague	0.5	2	8	>8	0.25	≤0.125	≤0.004	0.25	>8	>4	0.5	7	26	19	t586	E	E	-	CC5	-
B0047250	86	F	2018	Prague	0.5	2	8	>8	0.25	≤0.125	≤0.004	0.125	>8	>4	0.5	7	26	21	t2379	E	E	225	CC5	II
B0047286	78	M	2018	Olomouc	0.25	2	8	>8	0.125	≤0.125	≤0.004	0.125	>8	>4	0.5	8	28	19	t003	1	003	-	CC5	-
B0047335	66	F	2018	Pilsen	0.25	2	8	>8	1	≤0.125	≤0.004	0.125	>8	>4	0.5	6	26	20	t586	E	E	-	CC5	-
B0047338	61	M	2018	Pilsen	0.25	2	8	>8	0.5	≤0.125	≤0.004	0.125	>8	>4	0.5	6	29	21	t014	1	003	-	CC5	-
B0047341	66	M	2018	Pilsen	0.25	2	8	>8	0.25	≤0.125	≤0.004	0.125	>8	>4	0.5	6	27	20	t003	1	003	-	CC5	-
B0047366	30	M	2018	Liberec	0.25	2	8	>8	0.25	≤0.125	≤0.004	0.125	>8	>4	0.5	6	30	20	t014	1	003	225	CC5	II
B0047393	62	F	2018	South Bohemian	0.5	2	16	>8	0.25	≤0.125	0.008	0.25	>8	>4	0.5	7	31	20	t003	1	003	-	CC5	-
B0047401	66	M	2018	South Bohemian	0.5	2	8	>8	0.5	≤0.125	≤0.004	0.125	>8	>4	1	6	30	19	t586	E	E	-	CC5	-
B0047402	89	M	2018	South Bohemian	0.5	2	8	>8	0.5	≤0.125	0.008	0.125	>8	>4	0.5	6	10	20	t586	E	E	-	CC5	-
B0047418	42	F	2018	Usti nad Labem	0.5	2	8	>8	0.5	0.25	≤0.004	0.125	1	>4	1	17	7	21	t034	2	011	-	CC398	-
B0047443	79	F	2018	Prague	0.25	2	8	>8	0.25	≤0.125	≤0.004	0.125	>8	>4	1	7	28	22	t014	1	003	-	CC5	-
B0047455	60	M	2018	Prague	0.5	2	16	>8	0.5	0.25	≤0.004	0.125	>8	>4	1	7	30	19	t586	E	E	-	CC5	-
B0047500	62	F	2018	South Bohemian	0.25	2	8	>8	0.25	≤0.125	≤0.004	0.125	>8	>4	0.5	13	24	24	t003	1	003	-	CC5	-
B0047516	77	F	2018	Central Bohemian	0.25	2	8	>8	0.25	≤0.125	≤0.004	0.125	>8	>4	1	6	28	18	t586	E	E	-	CC5	-
B0047518	61	F	2018	Central Bohemian	0.25	2	8	>8	0.25	≤0.125	≤0.004	0.125	>8	>4	1	10	27	25	t586	E	E	-	CC5	-
B0047548	88	M	2018	Pilsen	0.5	4	16	>8	0.25	≤0.125	≤0.004	0.125	>8	>4	1	9	27	19	t014	1	003	-	CC5	-
B0047561	81	M	2018	Vysocina	0.25	2	8	>8	0.5	≤0.125	≤0.004	0.125	>8	>4	1	8	27	20	t003	1	003	-	CC5	-
B0047608	68	M	2018	Pardubice	0.25	2	8	>8	1	≤0.125	0.008	0.125	>8	**0.25**	1	9	27	19	t003	1	003	-	CC5	-
B0047641	72	M	2018	Prague	0.25	2	>64	>8	>8	≤0.125	≤0.004	0.125	>8	>4	1	6	27	19	t7657	1	003	-	CC5	-
B0047737	84	F	2018	Vysocina	0.25	2	8	>8	0.5	≤0.125	≤0.004	0.125	>8	>4	0.5	8	28	20	t003	1	003	-	CC5	-
B0047770	58	M	2018	Pardubice	0.25	2	8	>8	0.5	≤0.125	0.008	0.125	>8	>4	1	8	27	19	t014	1	003	-	CC5	-
B0047790	81	M	2018	Prague	0.25	2	8	>8	0.5	1	≤0.004	0.125	>8	>4	1	6	26	22	t003	1	003	-	CC5	-
B0047833	74	F	2018	Usti nad Labem	0.25	2	8	>8	0.5	≤0.125	0.008	0.125	>8	>4	0.5	6	30	17	t586	E	E	-	CC5	-
B0047873	50	F	2018	Karlovy Vary	0.25	2	8	>8	0.25	≤0.125	≤0.004	0.125	>8	>4	0.5	11	28	19	t045	1	003	-	CC5	-
B0047935	83	F	2018	Central Bohemian	0.25	2	8	>8	0.5	≤0.125	≤0.004	0.125	>8	>4	0.5	10	25	18	nt	-	-	-	-	-
B0047998	59	F	2018	Central Bohemian	0.5	2	8	>8	0.5	≤0.125	≤0.004	0.125	>8	>4	0.5	7	27	17	t586	E	E	-	CC5	-
B0047999	66	F	2018	Central Bohemian	0.5	2	16	>8	0.25	≤0.125	≤0.004	0.125	>8	>4	0.5	7	26	17	t586	E	E	-	CC5	-
B0048037	85	F	2018	Prague	0.25	2	8	>8	0.5	≤0.125	≤0.004	0.125	>8	>4	0.5	9	26	18	t003	1	003	-	CC5	-
B0048049	78	F	2018	Pilsen	0.25	2	8	>8	0.5	≤0.125	≤0.004	0.125	>8	>4	0.5	7	29	18	t003	1	003	-	CC5	-
B0048051	21	M	2018	South Moravian	0.5	2	8	1	>8	≤0.125	≤0.004	>4	0.5	0.25	1	18	10	20	t084	S	S	1535	CC15	V
B0048070	85	F	2018	South Bohemian	0.25	2	8	>8	0.5	≤0.125	≤0.004	0.125	>8	>4	0.5	8	27	19	t003	1	003	-	CC5	-
B0048099	73	F	2018	Prague	0.25	2	8	>8	0.25	≤0.125	≤0.004	0.125	>8	>4	0.5	6	31	19	t586	E	E	-	CC5	-
B0048151	58	M	2018	Prague	0.25	2	8	0.5	0.5	≤0.125	≤0.004	0.125	1	0.25	1	12	29	21	t359	6	NF	97	CC97	IV
B0048320	47	F	2018	Liberec	0.25	2	8	>8	0.5	≤0.125	≤0.004	0.125	>8	>4	0.5	10	25	20	t003	1	003	-	CC5	-
B0048363	79	F	2018	Pilsen	0.5	4	8	>8	0.25	≤0.125	>0.5	0.125	>8	>4	1	7	25	23	t893	1	003	-	CC5	-

B0048389	68	M	2018	Prague	0.25	2	8	>8	0.5	≤0.125	≤0.004	0.125	>8	>4	1	6	28	17	t586	E	E	-	CC5	-
B0048442	85	M	2018	South Moravian	0.25	2	8	>8	0.25	2	≤0.004	0.125	>8	>4	0.5	9	10	20	t045	1	003	-	CC5	-

male (M), female (F), non-typeable (nt), clonal complex (CC), sequence type (ST), excluded (E), singleton (S), no founder (NF), "-" (the isolate was not typed by the given method)

[1] antimicrobial susceptibility to tigecycline (TGC), linezoid (LNZ), chloramphenicol (CMP), ciprofloxacin (CIP), gentamicin (GEN), trimethoprim/sulfamethoxazole (SXT), rifampicin (RIF), fusidic acid (FUS), erythromycin (ERY), clindamycin (CLI), vancomycin (VAN), cefoxitine (FOX), tetracycline (TET) and ceftaroline (CPT) was tested either by microdilution or disc diffusion method

[2] inducible clindamycin resistance is highlighted in **bold**
