# Supplementary material for: Antibiotic Resistance, spa Typing and Clonal Analysis of Methicillin-Resistant Staphylococcus aureus (MRSA) Isolates from Blood of Patients Hospitalized in the Czech Republic
Source: Antibiotics (Basel). 2021 Apr 6;10(4):395. doi: 10.3390/antibiotics10040395 (PMC8067498; doi:10.3390/antibiotics10040395)
Supplement: Supplementary file 1 [file antibiotics-10-00395-s001.zip › Supplementary table S2.pdf]

Table S2: Primers used in the study.

		***mecA*/*mecC* detection**		
**Gene**	**Primer pair**	**Nucleotide Sequence (5'-3')**	**Size [bp]**	**Reference**
mecA	MECA P4	TCC AGA TTA CAA CTT CAC CAG G	162	[42]
	MECA P7	CCA CTT CAT ATC TTG TAA CG		
mecC	*mecA*$_{LGA251}$MultiFP	GAA AAA AAG GCT TAG AAC GCC TC	138	[43]
	mecA$_{LGA251}$MultiRP	GAA GAT CTT TTC CGT TTT CAG C		
		**SCC*mec* typing**		
**Primer specificity (SCC*mec* type, region)**	**Primer pair**	**Nucleotide Sequence (5'-3')**	**Size [bp]**	**Reference**
I, J1 region	CIF2 F2	TTC GAG TTG CTG ATG AAG AAG G	495	[47]
	CIF2 R2	ATT TAC CAC AAG GAC TAC CAG C		
V, *ccr* complex	ccrC F2	GTA CTC GTT ACA ATG TTT GG	449	
	ccrC R2	ATA ATG GCT TCA TGC TTA CC		
III, J3 region	RIF5 F10	TTC TTA AGT ACA CGC TGA ATC G	414	
	RIF5 R13	GTC ACA GTA ATT CCA TCA ATG C		
V, J1 region	SCCmec V J1 F	TTC TCC ATT CTT GTT CAT CC	377	
	SCCmec V J1 R	AGA GAC TAC TGA CTT AAG TGG		
I, II, IV, and VI, J3 region	dcs F2	CAT CCT ATG ATA GCT TGG TC	342	
	dcs R1	CTA AAT CAT AGC CAT GAC CG		
II and IV, *ccr* complex	ccrB2 F2	AGT TTC TCA GAA TTC GAA CG	311	
	ccrB2 R2	CCG ATA TAG AAW GGG TTAG C		
II, J1 region	kdp F1	AAT CAT CTG CCA TTG GTG ATG C	284	
	kdp R1	CGA ATG AAG TGA AAG AAA GTG G		
III, J1 region	SCCmec III J1 F	CAT TTG TGA AAC ACA GTA CG	243	
	SCCmec III J1 R	GTT ATT GAG ACT CCT AAA GC		
II and III, *mec* complex	mecI P2	ATC AAG ACT TGC ATT CAG GC	209	
	mecI P3	GCG GTT TCA ATT CAC TTG TC		
Internal positive control	mecA P4	TCC AGA TTA CAA CTT CAC CAG G	162	
	mecA P7	CCA CTT CAT ATC TTG TAA CG		
II	Type II-F	CGT TGA AGA TGA TGA AGC G	398	[48]
	Type II-R	CGA AAT CAA TGG TTA ATG GAC C		
V	Type V-F	GAA CAT TGT TAC TTA AAT GAG CG	325	
	Type V-R	TGA AAG TTG TAC CCT TGA CAC C		
		**Multilocus sequence typing**		
**Gene**	**Primer pair**	**Nucleotide Sequence (5'-3')**		**Reference**
Carbamate kinase (*arcC*)	*arcC*-Up	TTG ATT CAC CAG CGC GTA TTG TC		[46]
	arcC-Dn	AGG TAT CTG CTT CAA TCA GCG		
Shikimate dehydrogenase (*aroE*)	*aroE*-Up	ATC GGA AAT CCT ATT TCA CAT TC		
	aroE-Dn	GGT GTT GTA TTA ATA ACG ATA TC		
Glycerol kinase (*glpF*)	*glpF*-Up	CTA GGA ACT GCA ATC TTA ATC C		
	glpF-Dn	TGG TAA AAT CGC ATG TCC AAT TC		
Guanylate kinase (*gmk*)	*gmk*-Up	ATC GTT TTA TCG GGA CCA TC		
	gmk-Dn	TCA TTA ACT ACA ACG TAA TCG TA		
Phosphate acetyltransferase (*pta*)	*pta*-Up	GTT AAA ATC GTA TTA CCT GAA GG		
	pta-Dn	GAC CCT TTT GTT GAA AAG CTT AA		
Triosephosphate isomerase (*tpi*)	*tpi*-Up	TCG TTC ATT CTG AAC GTC GTG AA		
	tpi-Dn	TTT GCA CCT TCT AAC AAT TGT AC		
Acetyl coenzyme A acetyltransferase (*yqiL*)	*yqiL*-Up	CAG CAT ACA GGA CAC CTA TTG GC		
	yqiL-Dn	CGT TGA GGA ATC GAT ACT GGA AC		

spa typing			
**Primer specificity**	**Primer pair**	**Nucleotide Sequence (5'-3')**	**Reference**
Staphylococcus protein A (*spa*) repeat region	spa-1113f	TAA AGA CGA TCC TTC GGT GAG C	[44]
	spa-1514r	CAG CAG TAG TGC CGT TTG CTT	
